# Design and Synthesis of Chiral Zn^2+^ Complexes Mimicking Natural Aldolases for Catalytic C–C Bond Forming Reactions in Aqueous Solution

**DOI:** 10.3390/ijms15022087

**Published:** 2014-01-29

**Authors:** Susumu Itoh, Shotaro Sonoike, Masanori Kitamura, Shin Aoki

**Affiliations:** 1 Production Technology Laboratories, Kaken Pharmaceutical Co., LTD, 301 Gensuke, Fujieda, Shizuoka 426-8646, Japan; E-Mail: itou_susumu@kaken.co.jp; 2 Faculty of Pharmaceutical Sciences, Tokyo University of Science, 2641 Yamazaki, Noda, Chiba 278-8510, Japan; E-Mails: sonoike.shotaro51@chugai-pharm.co.jp (S.S.); kitamura@p.kanazawa-u.ac.jp (M.K.); 3 Center for Technologies against Cancer, Tokyo University of Science, 2641 Yamazaki, Noda, Chiba 278-8510, Japan

**Keywords:** chemoenzymatic synthesis, enzyme mimics, zinc, asymmetric synthesis, cofactor regeneration

## Abstract

Extending carbon frameworks via a series of C–C bond forming reactions is essential for the synthesis of natural products, pharmaceutically active compounds, active agrochemical ingredients, and a variety of functional materials. The application of stereoselective C–C bond forming reactions to the one-pot synthesis of biorelevant compounds is now emerging as a challenging and powerful strategy for improving the efficiency of a chemical reaction, in which some of the reactants are subjected to successive chemical reactions in just one reactor. However, organic reactions are generally conducted in organic solvents, as many organic molecules, reagents, and intermediates are not stable or soluble in water. In contrast, enzymatic reactions in living systems proceed in aqueous solvents, as most of enzymes generally function only within a narrow range of temperature and pH and are not so stable in less polar organic environments, which makes it difficult to conduct chemoenzymatic reactions in organic solvents. In this review, we describe the design and synthesis of chiral metal complexes with Zn^2+^ ions as a catalytic factor that mimic aldolases in stereoselective C–C bond forming reactions, especially for enantioselective aldol reactions. Their application to chemoenzymatic reactions in aqueous solution is also presented.

## 1. Introduction

C–C bond formation is one of the fundamental transformations in organic synthesis. Extension of a carbon framework via a series of C–C bond forming reactions is essential to the synthesis of natural products, pharmaceutically active compounds, active agrochemical ingredients, and related functional materials [1]. One of most important applications of C–C bond forming reactions is one-pot synthesis, whereby reactants are subjected to successive chemical reactions in just one reactor. This methodology is convenient not only in the laboratory, but also in industrial reactions, because lengthy separation and purification processes of the intermediates can be avoided, resulting in time and resource-saving and, eventually, in a more efficient chemical synthesis. Despite the remarkable progress achieved in one-pot multistep synthetic methodologies including enantioselective C–C bond formation in organic solvents, only a few attempts have been made to combine a chemical catalyst and a biocatalyst in a one-pot multistep process, especially in water-containing solvent systems [2].

**Scheme 1.**
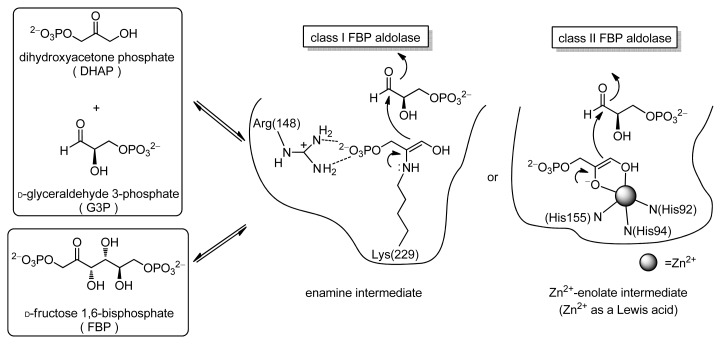
Proposed mechanism for aldolase-catalyzed reactions.

It is clear that much can be learned from natural enzymes for the design of water-soluble asymmetric catalysts. Typical examples of enzymes that catalyze C–C bond forming reactions in living systems would be aldolases, a class of enzymes that accelerate aldol and retro-aldol reactions in a stereospecific and reversible manner in natural metabolic pathways [3]. For example, fructose 1,6-bis(phosphate) aldolase (FBP-aldolase, EC 4.1.2.13) catalyzes the cleavage of d-fructose 1,6-bis(phosphate) (FBP) to give dihydroxyacetone phosphate (DHAP) and d-glyceraldehyde 3-phosphate (G3P), as well as the reverse formation of FBP from DHAP and G3P (Scheme 1). Natural aldolases can be classified into two groups on the basis of their reaction mechanisms. In the case of class I aldolases, an enamine intermediate is formed between the lysine residue of the enzyme and the carbonyl group of the substrate. In class II aldolases, a zinc(II) ion cofactor acts as a Lewis acid to generate enolates at the active site.

**Scheme 2.**
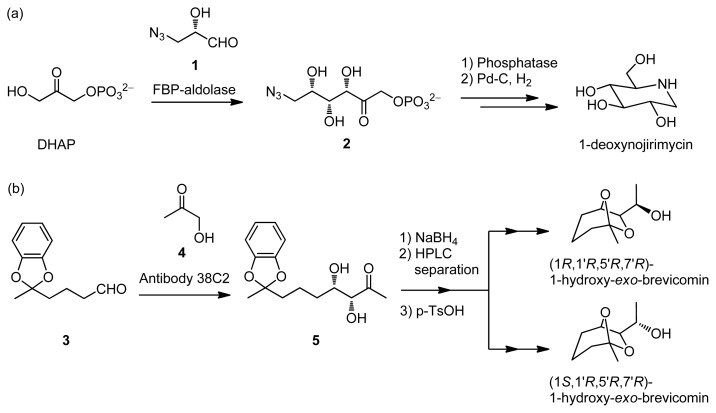
Examples of aldol reactions using (**a**) FBP-aldolase and (**b**) aldolase antibody 38C2.

The use of aldolases in organic and bio-organic synthesis has been found to be an effective method for producing aldol products with high stereoselectivities in aqueous solution [4]. Wong *et al*. reported on a short step synthesis of 1-deoxynojirimycin, a glycosidase inhibitor, in which aldol reaction between DHAP and **1**, using FBP-aldolase is conducted to obtain the important intermediate, **2** (Scheme 2a) [5]. In addition, Lerner and co-workers developed catalytic antibodies, which were obtained after screening of polyclonal antibodies for binding with the hapten [6,7]. The aldolase antibodies were found to catalyze aldol reactions by the enamine mechanism, analogous to class I aldolases. The aldol reaction between **3** and hydroxyacetone (HA, **4**), catalyzed by aldolase antibody 38C2, has been applied in highly enantioselective total syntheses of (1*R*, 1′*R*, 5′*R*, 7′*R*)- and (1*S*, 1′*R*, 5′*R*, 7′*R*)-1-hydroxy-*exo*-brevicomin (Scheme 2b) [8].

**Scheme 3.**
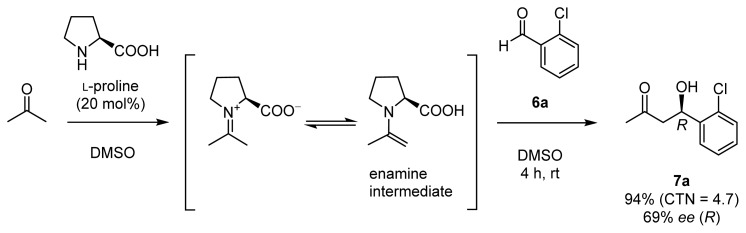
Direct asymmetric aldol reaction catalyzed by l-proline.

Chiral organocatalysts have recently emerged as a reagent class representing a new methodology for stereoselective aldol reactions, in that they are capable of mimicking class I aldolases [9]. In 2000, List, Lerner, and Barbas reported that l-proline serves as a catalyst for direct aldol reactions of acetone and benzaldehyde derivatives **6** to give aldol adducts such as **7** in good chemical yields and with moderate enantioselectivities (Scheme 3, CTN stands for catalytic turnover number) [10]. It was suggested that l-proline forms an enamine intermediate that react with aldehydes to give aldol products [11].

**Scheme 4.**
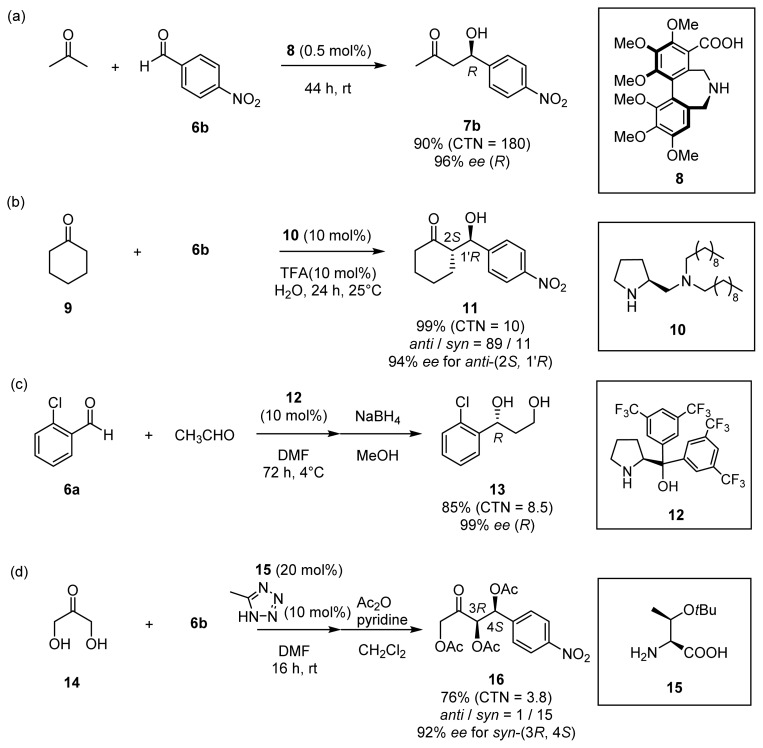
Examples of direct asymmetric aldol reactions catalyzed by organocatalysts ((**a**) **8**; (**b**) **10**; (**c**) **12**; and (**d**) **15**).

As the direct aldol reaction catalyzed by l-proline was first reported, a variety of organocatalysts for aldol reactions have been reported [12]. Representative examples are shown in Scheme 4. Maruoka and co-workers successfully extended the concept of amino acid catalysis to novel catalysts containing a binaphthyl or biphenyl axial chirality (e.g., **8**) [13]. The use of only 0.1 mol % of **8** in acetone afforded aldol products such as **7b** with high yields and enantioselectivities (Scheme 4a). Barbas *et al.* reported on asymmetric aldol reactions between cyclohexanone **9** and benzaldehydes **6** in water catalyzed by a combination of a lipophilic diamine **10** and trifluoroacetic acid (TFA) [14] (Scheme 4b). Hayashi’s group developed a proline-based catalyst, diarylprolinol **12**, for direct crossed-aldol reactions of acetaldehyde in DMF [15] (Scheme 4c). Barbas *et al*. performed *syn*-selective aldol reactions of unmodified dihydroxyacetone (DHA, **14**) catalyzed by O-*t*Bu-l Threonine **15** to yield acetylated triol products such as **16** [16] (Scheme 4d).

**Scheme 5.**
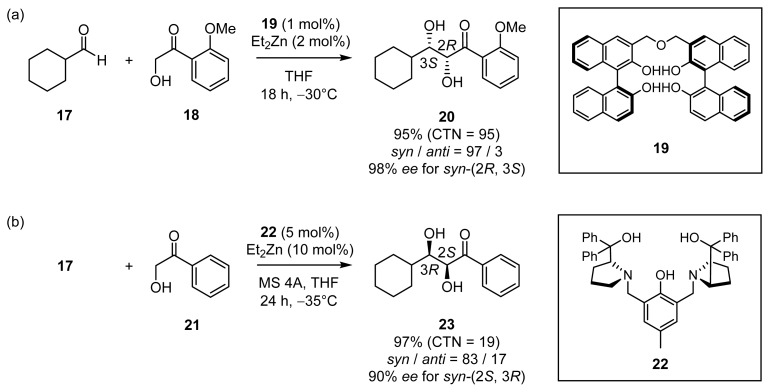
Examples of direct asymmetric aldol reactions catalyzed by chiral Zn^2+^ catalysts ((**a**) **19** and (**b**) **22**).

A number of excellent studies on chiral metal catalysts for stereoselective aldol reactions have also been reported [17]. Examples of Zn^2+^ catalysts for direct asymmetric aldol reactions include Et_2_Zn/linked BINOL **19** developed by Shibasaki [18], Trost’s Zn^2+^-semi crown ether **22** [19], and so forth, which can be considered as class II aldolase mimics functioning in organic solvents (Scheme 5). Both catalysts showed high catalytic activities on α-hydroxyketones (**18** and **21**) by dinuclear Zn^2+^ coordination sites for the enolate of α-hydroxyketones and aldehydes.

**Scheme 6.**
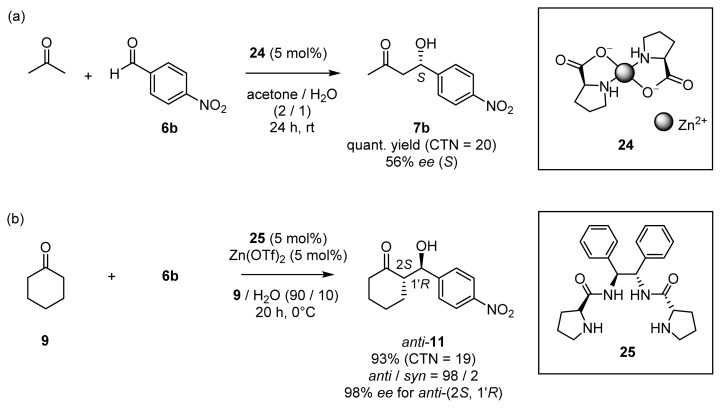
Examples of direct asymmetric aldol reactions catalyzed by chiral Zn^2+^ catalysts ((**a**) **24** and (**b**) **25**).

To date, natural and artificial catalysts that possess both functionalities, *i.e.*, Schiff-base forming part and Zn^2+^ site, have scarcely been reported. Some Zn^2+^ complexes of proline derivatives have been demonstrated to have the ability to catalyze direct asymmetric aldol reactions in aqueous media, thus mimicking class I and class II aldolases (Scheme 6). Reymond, Darbre, *et al.* showed that the 1:2 complex of Zn^2+^ and l-proline **24** (Zn(l-Pro)_2_) catalyzes aldol reactions of acetone and dihydroxyacetone **14** in aqueous system [20,21]. The use of 5 mol % of **24** gave aldol products from acetone and **6b** in quantitative yields, with 56% *ee* (*R*), at room temperature (Scheme 6a). They suggested that in the aldol reactions of these substrates, **24** forms enamine species with acetone, but that it forms Zn^2+^-enolate intermediates with **14**. Mlynarski’s group reported on Zn^2+^ complexes of a *C*_2_-symmetric chiral ligands containing two amino acid units, such as **25** [22]. These catalysts showed high reactivities and enantioselectivities in catalytic aldol reactions between acetone, cyclohexanone **9**, and hydroxyacetone **4** and several aldehydes in aqueous systems (up to 99% *ee*) (Scheme 6b).

**Scheme 7.**
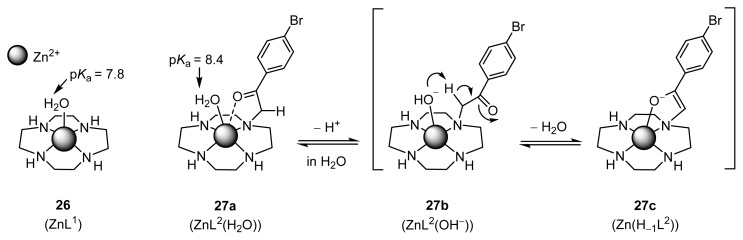
Zn^2+^ complexes as model for Zn^2+^ enzymes.

Meanwhile, it has been well established by Kimura and co-workers that a Zn^2+^ complex of cyclen **26** (ZnL^1^; cyclen = 1,4,7,10-tetraazacyclododecane or [12]aneN_4_) is a good model for the naturally occurring Zn^2+^ enzymes (Scheme 7) [23,24]. A Zn^2+^ complex of 4-bromophenacyl-pendant cyclen **27** (ZnL^2^), for example, was reported to be a good model for the class II aldolases [25]. The potentiometric pH titration of **27a** (ZnL^2^(H_2_O)) and spectroscopic data indicated that the Zn^2+^-bound HO^−^ in **27b** (ZnL^2^(OH^−^)) facilitated the deprotonation of the carbonyl α-proton of the phenacyl side chain to give the Zn^2+^-enolate form **27c** (Zn(H_−1_L^2^)) in aqueous solution. Note that Zn^2+^ ions in Zn^2+^-cyclen complexes exhibit strong Lewis acidity and that the p*K*_a_ values of the Zn^2+^-bound water molecules in **26** and **27** are 7.8 and 8.4, respectively.

With the backgrounds described above, we became interested in the reactivities and enantioselectivities of novel asymmetric catalysts, each dual-functionalized with an enamine-forming amino group and a Zn^2+^ complex of macrocyclic polyamines such as cyclen ([12]aneN_4_) or [15]aneN_5_. The development of enantioselective aldol reactions was envisaged, as they are one of the most important C–C bond forming reactions for producing β-hydroxy carbonyl compounds bearing two new stereogenic centers at the α- and β-positions of the carbonyl groups [17,26,27]. It should be noted that these reactions are performed mainly in aqueous solvents, which are considered to have enormous potential as a reaction mediums and are critical for chemoenzymatic reactions [28–30]. One of the advantages of these aldol reactions is that they would be applicable to the one-pot synthesis of biorelevantly important compounds by combination with enzymatic reactions. These results are reviewed below.

## 2. Results and Discussion

### 2.1. Chiral Catalysts that Are Dually Functionalized with Amino Acid and Zn^2+^ Complex Components for Direct Enantioselective Aldol Reactions Inspired by Natural Aldolases

#### 2.1.1. Design and Synthesis of Chiral Zn^2+^ Complexes

**Figure 1.**
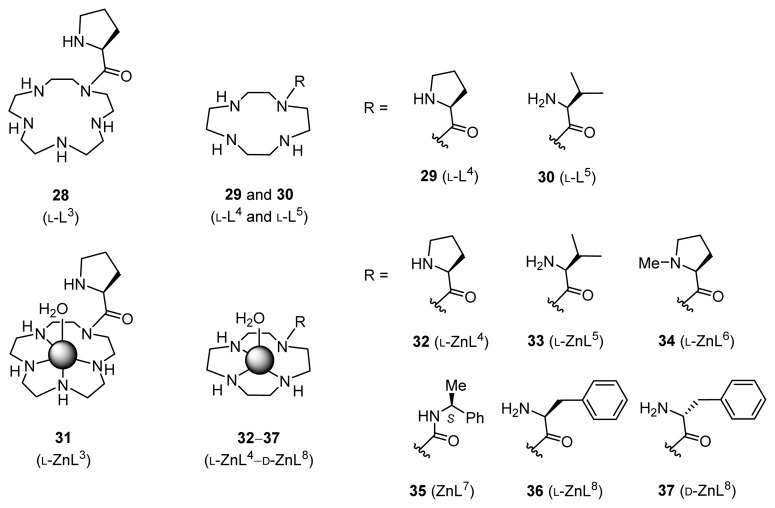
Zn^2+^ complexes for direct asymmetric aldol reactions.

The initially designed and synthesized Zn^2+^ complexes for stereoselective direct aldol reactions include **31** (l-ZnL^3^ prepared from l-prolyl-pendant [15]aneN_5_ **28** (l-L^3^)), **32** (l-ZnL^4^, prepared from l-prolyl-pendant cyclen **29** (l-L^4^)), and **33** (l-ZnL^5^ from l-valyl-pendant cyclen **30** (l-L^5^)), and the related complexes **34** (l-ZnL^6^), **35** (ZnL^7^), **36** (l-ZnL^8^), and **37** (d-ZnL^8^) (Figure 1). The ligands for Zn^2+^ complexes **31**–**37** (l-ZnL^3^–d-ZnL^8^) were synthesized from tetrakis(*tert*-butyloxycarbonyl)-[15]aneN_5_ (4-Boc-[15]aneN_5_) or tris(*tert*-butyloxycarbonyl)-[12]aneN_4_ (3-Boc-[12]aneN_4_) with *N*-protected amino-acid derivatives [31]. The Zn^2+^ complexes were prepared *in situ* immediately prior to use by reacting the acid-free ligands with Zn^2+^ ions.

#### 2.1.2. Complexation Properties of Chiral Zn^2+^ Complexes

**Scheme 8.**
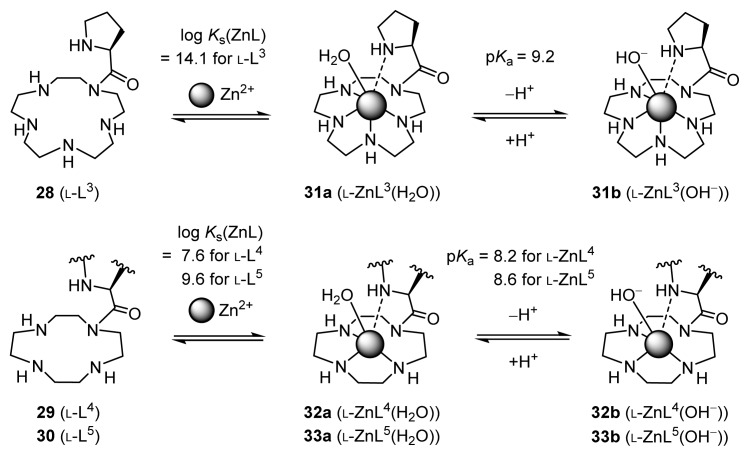
Equilibria for Zn^2+^ complexation of **28** (l-L^3^), **29** (l-L^4^), and **30** (l-L^5^).

Potentiometric pH titration is a general and reliable method to determine the deprotonation constants (*K*_ai_ defined by Equation (1)) and the metal complexation constants (Zn^2+^ complexation constants, *K*_s_(ZnL), as defined by Equation (2) in this work) of given ligands in aqueous solution. Analysis of typical potentiometric pH titration curves for 0.5 mM ligands (l-H_5_L^3^, l-H_4_L^4^, and l-H_4_L^5^) and for mixtures of 0.5 mM ligands **31**–**33** (l-H_5_L^3^, l-H_4_L^4^, and l-H_4_L^5^), plus 0.5 mM ZnSO_4_ gave complexation constants, log *K*_s_(ZnL), and p*K*_a_(ZnL) values of Zn^2+^-bound water in Zn^2+^ complexes (defined by Equation (3)), as summarized in Scheme 8 [31].

(1) $${\text{H}}_{\left(5-i\right)}\text{L}⇌{\text{H}}_{\left(4-i\right)}\text{L}+{\text{H}}^{+}\;\;\;:K_{\text{a}i}={\left[{\text{H}}_{\left(4-i\right)}\text{L}\right]}_{a\text{H}+}/[{\text{H}}_{\left(5-i\right)}\text{L}]\;(i=1-5)$$

(2) $$\text{L}+\text{Zn}⇌\text{ZnL }\;\;:K_{\text{s}}(\text{ZnL})=[\text{ZnL}]/[\text{L}][{\text{Zn}}^{2+}]\;({\text{M}}^{-1})$$

(3) $$\text{ZnL}({\text{H}}_{2}\text{O})⇌\text{ZnL}({\text{OH}}^{-})+{\text{H}}^{+}\;\;\;:K_{\text{a}}(\text{ZnL})={\left[\text{ZnL}({\text{OH}}^{-})\right]}_{a\text{H}+}/[\text{ZnL}({\text{H}}_{2}\text{O})]$$

**Scheme 9.**
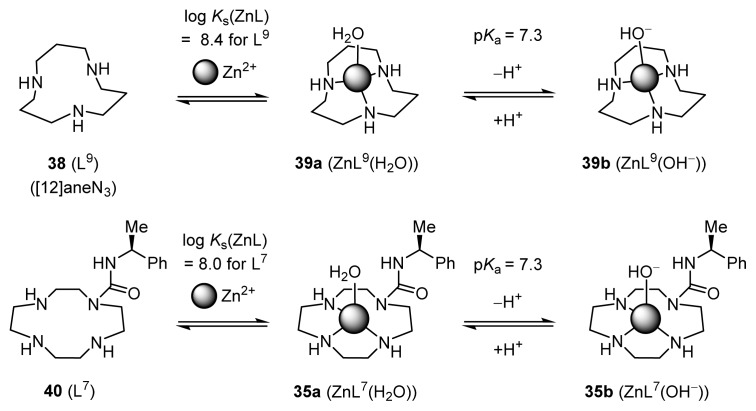
Equilibria for Zn^2+^ complexation of **38** (L^9^) and **40** (L^7^).

The log *K*_s_(ZnL) for **28** (L^3^) is 14.1 (Scheme 8), which is much greater than the corresponding values for [12]aneN_3_ **38** (L^9^, 8.4) [32] and **40** (L^7^, 8.0) [31], which have been established as tridentate ligands for Zn^2+^ (Scheme 9). The deprotonation constant for the Zn^2+^-bound water of l-ZnL^3^ (for **31a** ⊂ **31b**) (p*K*_a_(ZnL)) was calculated to be 9.2, which is greater than that for Zn^2+^-cyclen complex **26** (ZnL^1^) of 7.8 in Scheme 7. These data suggest that **28** (l-L^3^) serves as a tetradentate or pentadentate ligand to Zn^2+^. For example, **31** (l-ZnL^3^) (**31a** (l-ZnL^3^(H_2_O)) + **31b** (l-ZnL^3^(OH^−^))) is formed almost quantitatively at pH 7.4 in a mixture of 25 mM l-L^3^ and 25 mM Zn^2+^.

In addition, as shown in Scheme 8, the p*K*_a_(ZnL) values for the Zn^2+^-bound water in **31**–**33** (l-ZnL^3^– l-ZnL^5^) were found to be 9.2, 8.2, and 8.6, respectively, which are larger than those for **26** (ZnL^1^) of 7.8 and **39** (ZnL^9^) of 7.3. These data indicate that the Lewis acidity of Zn^2+^ in **31**–**33** (l-ZnL^3^– l-ZnL^5^) is weaker than that in **26** (ZnL^1^) and **39** (ZnL^9^). The fact that the p*K*_a_(ZnL) values for **32** (8.2) and **33** (8.6) are somewhat larger than those of **35** (7.3) and **39** (7.3), which are Zn^2+^ complexes of tridentate ligands, suggested the possibility that the nitrogen components in the prolyl and valyl units of **32** and **33** coordinate only weakly to Zn^2+^ (the cyclen units of these ligands are tridentate binders for Zn^2+^).

#### 2.1.3. Enantioselective Aldol Reactions in Aqueous Media Catalyzed by Chiral Zn^2+^ Complexes

On the basis of the data on the Zn^2+^ complexation behavior of the chiral ligands, described above, the aldol reaction between acetone and 2-chlorobenzaldehyde **6a** in the presence of the chiral catalysts in DMSO/acetone or acetone/H_2_O systems was examined. The results are summarized in Table 1. Most of the reactions in Table 1 were carried out at 37 °C as an enzyme model study.

As described in the Introduction, l-proline (20 mol % relative to the aldehyde) gave **7a** with a good chemical yield (85%) and enantioselectivity (67% *ee* (*R*)) in DMSO/acetone at 25 °C (entry 1). As listed in entries 2 and 3, **31** (l-ZnL^3^) and **32** (l-ZnL^4^) gave **7a** with low yields and 1% *ee* (*R*) and 34% *ee* (*R*), respectively ([L] = [Zn^2+^] = 10 mM for quantitative formation of Zn^2+^ complexes, based on the results of potentiometric pH titrations).

In the acetone/H_2_O system, l-proline gave **7a** in 22% yield and with 48% *ee* (*R*) at 37 °C (entry 4), both of which were lower than those in the DMSO/acetone system (entry 1). In contrast, **31** (l-ZnL^3^) gave better yield (43% yield with a nearly racemic adduct) at 37 °C (entry 5) than in DMSO/acetone (entry 2). Interestingly, **32** (l-ZnL^4^) gave **7a** in good yield (73%) and enantioselectivity (80% *ee* (*R*)) in entry 6. Metal-free **29** (l-L^4^) gave only the racemic aldol product (entry 7), whereas the Cd^2+^ and Cu^2+^ complexes of l-L^4^ (**41a** and **41b**) promoted aldol reactions only to a negligible extent (entries 8 and 9). It had previously been reported that the Lewis acidity of the Zn^2+^-cyclen complex is higher than that of Cd^2+^-cyclen [33]. We therefore concluded that the Lewis acidity of Zn^2+^ is an important factor for this enantioselective aldol reaction.

Other Zn^2+^ complexes were also tested, as listed in entries 10–16. Zn^2+^ complex **33** (l-ZnL^5^), which contains a valine unit, gave results almost the same as those obtained with **32** (l-ZnL^4^) (entry 10). The Zn^2+^ complexes of *N*-methylprolyl-pendant cyclen **34** (l-ZnL^6^) and (*S*)-methylbenzylurenyl-pendant cyclen **35** (ZnL^7^) were used for comparison. Although it is unlikely that the tertiary amine on the side chain of **34** (l-ZnL^6^) would form a Schiff-base with acetone, **34** (l-ZnL^6^) gave **7a** in moderate yield but with low *ee* (entry 11). On the other hand, **35** (ZnL^7^), having no amino group, scarcely yielded the aldol adduct (entry 12). **36** (l-ZnL^8^) and **37** (d-ZnL^8^), which contained l- and d-phenylalanyl side chains, respectively, gave **7a** in somewhat higher chemical yields and enantioselectivities (91% *ee* (*R*) with **36** (l-ZnL^8^) and 91% *ee* (*S*) with **37** (d-ZnL^8^)) than those for **32** (l-ZnL^4^) and **33** (l-ZnL^5^) (entries 6 and 10 *versus* 13 and 14). Zn^2+^-cyclen **26** (ZnL^1^) alone, in which the Zn^2+^ is coordinated by four nitrogen atoms, and a combination of l-proline and **26** (ZnL^1^) showed negligible catalytic activity (entries 15 and 16).

These results suggest that primary or secondary amino groups on the side chains are important and that the amino acid portions of **31**–**33** (l-ZnL^3^– l-ZnL^5^), **36** (l-ZnL^8^) and **37** (d-ZnL^8^), function as bases for the deprotonation of acetone activated by Zn^2+^, rather than as Schiff-base forming units.

Aldol reactions between acetone and benzaldehyde derivatives **6b**–**e** were carried out in acetone/H_2_O (4/1) in the presence of 50 mM ZnL catalysts (Table 2). When 4-nitrobenzaldehyde **6b** was used as an acceptor in the presence of **32** (l-ZnL^4^) and **33** (l-ZnL^5^) (5 mol %), the product **7b** was obtained in 63% *ee* (*R*) and 86% *ee* (*R*), respectively (entries 1 and 2). The use of 10 mol % of **36** (l-ZnL^8^) and **37** (d-ZnL^8^) gave the corresponding product **7b** and **7c** in almost quantitative yields with 90% *ee* (entries 4–6). Whereas the reactivities of 4-bromobenzaldehyde **6d** and 2-naphthylaldehyde **6e** were somewhat lower than **6b**, **7d**, and **7e** were obtained in good enantioselectivities in the presence of **33** (l-ZnL^5^) (entries 8 and 10).

#### 2.1.4. Fine Tuning of Structure of Chiral Zn^2+^ Complexes

**Figure 2.**
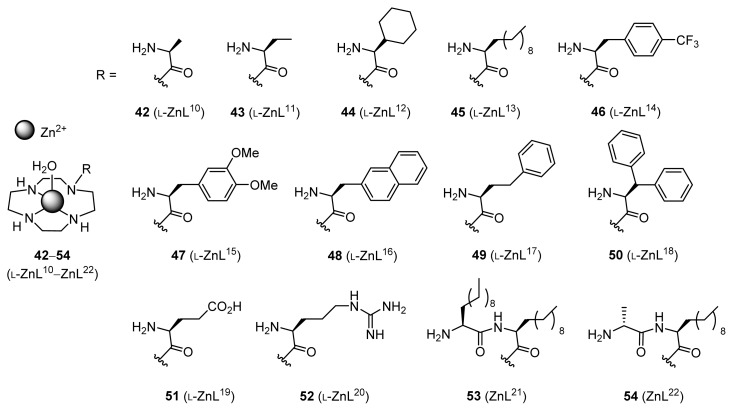
Structures of Zn^2+^ complexes containing aliphatic, aromatic, anionic, cationic, and dipeptide side chains.

In order to improve the aldol reaction between acetone and benzaldehyde derivatives, various Zn^2+^ complexes containing aliphatic, aromatic, anionic, cationic, and dipeptide side chains, **42**–**54** (l-ZnL^10^–ZnL^22^), were synthesized and their activity examined (Figure 2).

The aldol reaction between acetone and 2-chlorobenzaldehyde **6a** was performed in acetone/H_2_O (4/1 or 9/1) in the presence of 50 mM Zn^2+^ complexes. The results are summarized in Table 3.

In entry 1, **42** (l-ZnL^10^), which contains alanine as the amino-acid unit, afforded **7a** in high optical yield. In addition, **43** (l-ZnL^11^), **44** (l-ZnL^12^), and **45** (l-ZnL^13^), containing aliphatic side chains exhibited higher catalytic activities than **32** (l-ZnL^4^), **33** (l-ZnL^5^), and **36** (l-ZnL^8^) in Table 2 (entries 2–4). **46** (l-ZnL^14^) and **47** (l-ZnL^15^), which contained 4-trifluoromethylphenylalanine and 3,4-dimethoxyphenylalanine as amino-acids, respectively, afforded lower optical yields than **36** (l-ZnL^8^), which contained a phenylalanine group (entries 5 and 6). In addition, **48** (l-ZnL^16^) and **49** (l-ZnL^17^), which contained aromatic side chains, also afforded higher catalytic activities than **32** (l-ZnL^4^), **33** (l-ZnL^5^), and **36** (l-ZnL^8^) (entries 7 and 8). On the other hand, **50** (l-ZnL^18^), having a bulky diphenylmethyl group, gave **7a** in moderate yield and 88% *ee* (*R*) (entry 9). These results suggest that Zn^2+^ complexes that contain sufficiently bulky side chains, such as decyl, naphthyl, and phenylethyl groups, are expected to have efficient catalytic activity. In entries 10 and 11, **51** (l-ZnL^19^), which contains an anionic propanoate side chain (from Glu), and **52** (l-ZnL^20^), which contains a cationic guanidium group (from Arg), afforded **7a** in 78% *ee* (*R*) and 92% *ee* (*R*), respectively, thus, indicating negligible electrostatic effects on the enantioselectivity of the products. It was possible to improve the *ee* values by increasing the amount of acetone and performing the reaction at a lower temperature (entries 12–14 *versus* 2, 4 and 8, respectively).

Next, **53** and **54** (ZnL^21^ and ZnL^22^), which contained dipeptide side chains, were prepared based on the assumption that the presence of more hydrophobic and hydrogen bonding functionalities around the Zn^2+^ site would improve their catalytic activity. However, these Zn^2+^ complexes resulted in low chemical and optical yields (entries 15 and 16), thus, suggesting that one amino-acid side chain is suitable for aldol reactions catalyzed by the ZnL series.

Table 4 summarizes the aldol reactions between acetone and various benzaldehydes (**6b**–**c** and **6f**–**g**), as catalyzed by **45** (l-ZnL^13^) and **49** (l-ZnL^17^) in acetone/H_2_O (9/1). When 4-chlorobenzaldehyde **6c** was used, **7c** was obtained in good yield and 94% *ee* (*R*) and 95% *ee* (*R*), respectively (entries 3 and 4) and, when 4-, 3-, and 2-nitrobenzaldehydes (**6b**, **6f**, and **6g**) were used as acceptors, high chemical and optical yields were similarly observed (entries 1, 2, and 5–8).

#### 2.1.5. UV Titrations of Acetylacetone (Acac) with l-Proline, l-Valine, Zn(OTf)_2_, and Zn^2+^ Complexes (**32** (l-ZnL^4^), **33** (l-ZnL^5^), **35** (ZnL^7^), and **26** (ZnL^1^)) in a Mechanistic Study

In order to examine the issue of whether amino groups of l-proline, **32** (l-ZnL^4^), and **33** (l-ZnL^5^) form Schiff-bases with substrates, we initially carried out a UV titration of acetone with l-proline and **32** (l-ZnL^4^). However, the UV spectral change was negligible.

As described in the Introduction, it was suggested that the mechanism of the aldolase antibodies involves enamine formation as the result of a reaction between the ɛ-amino group of Lys and the ketone substrate [7]. In this scenario, it was reported that enaminone between the Lys residue of the antibody and β-diketone exhibit a strong UV absorption at 316 nm (ɛ_316_ ~ 15,000 M^−1^·cm^−1^).

**Figure 3.**
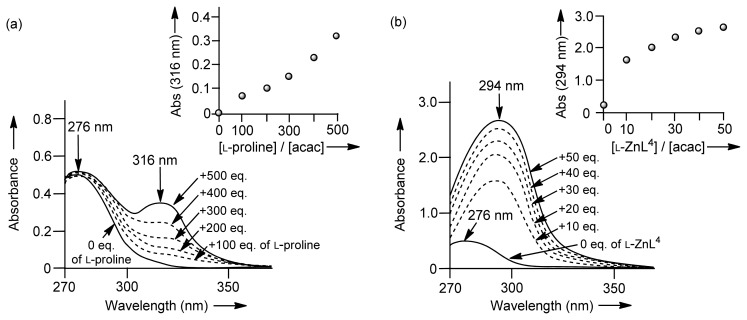
(**a**) Change in the UV spectra of 0.2 mM acac upon addition of l-proline (0–500 equiv) in DMSO (dimethyl sulfoxide)/H_2_O (1/2) at 25 °C; (**b**) Change in UV spectra of 0.2 mM acetylacetone (acac) with **32** (l-ZnL^4^) upon addition of (0–50 equiv) in DMSO/H_2_O (1/2) at 25 °C.

**Scheme 10.**
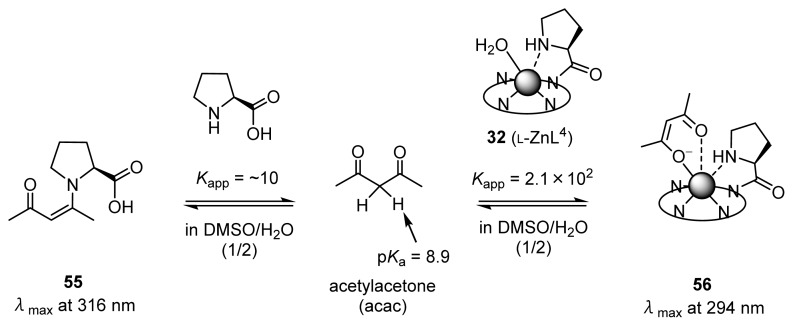
Equilibria of acetylacetone (acac) with L-proline or **32** (l-ZnL^4^).

Then, UV titrations of acetylacetone (acac) with l-proline and chiral Zn^2+^ complexes were conducted (Scheme 10). Figure 3a shows the results of the UV absorption titration of acac (0.2 mM) with l-proline in DMSO/H_2_O (1/2) at 25 °C, in which the absorption maxima of acac shifted from 276 to 316 nm at increasing concentrations of l-proline (0–500 equiv). These results suggest the formation of a Schiff-base between l-proline and acac. On the other hand, the absorption maxima of acac was shifted from 276 to 294 nm upon the addition of **32** (l-ZnL^4^) (0–50 equiv), as shown in Figure 3b. In order to assign the strong UV absorption at 294 nm shown in Figure 3b, the change in the UV spectra of acac as a function of pH in DMSO/H_2_O (1/2) was measured. The deprotonation constant of the carbonyl α-proton of acac was determined to be 8.9 by potentiometric pH titrations in H_2_O with *I* = 0.1 (NaNO_3_) at 25 °C. The UV absorption spectra of acac at pH 4.1 and 7.3 having a λ_max_ (absorption maxima) at 276 nm shifted to 293 nm upon the addition of OH^−^ (pH 9.8), possibly corresponds to the monoanionic form of acac ((acac)^−^).

These results suggest that l-proline forms the enaminone **55** with acac (λ_max_ = 316 nm), while **32** (l-ZnL^4^) induces the formation of the 1:1 l-ZnL^4^-(acac)^−^ complex **56** (λ_max_ = 294 nm) (Scheme 10). Analysis of the titration curve in the inset of Figure 3a with the aid of the software program “BIND WORKS” gave an enaminone formation constant (*K*_app_) of *ca.* ~10 M^−1^ for acac with l-proline. In DMSO/H_2_O (95/5), the formation constant for l-proline-acac enaminone **55** was increased to 62 M^−1^, indicating that these equilibria are solvent-dependent. On the other hand, an analysis of the titration curve in the inset of Figure 3b gave a complexation constant for **56** of 2.1 × 10^2^ M^−1^ in DMSO/H_2_O (1/2).

The apparent formation constants of enaminone from acac with l-valine (0–500 equiv) or ZnL-(acac)^−^ complexes (*K*_app_) from Zn(OTf)_2_ (0–50 equiv), **33** (l-ZnL^5^) (0–50 equiv), **35** (ZnL^7^) (0–20 equiv), and **26** (ZnL^1^) (0–20 equiv), are summarized in Table 5. Upon addition of l-valine, the absorption maxima shifted from 276 to 316 nm, similarly to what was observed when l-proline was mixed with acac. On the other hand, Zn(OTf)_2_ showed an absorption maximum of 289 nm, similarly to what was observed for a mixture of **32** (l-ZnL^4^) and acac. The Zn^2+^ complexes **33** (l-ZnL^5^) and **35** (ZnL^7^) also showed absorption maxima at 292–295 nm. Interestingly, the change in the UV spectra of acac upon the addition of **26** (ZnL^1^), in which Zn^2+^ is 4-coordinated to four nitrogen atoms, was negligible. These results suggest that **33** (l-ZnL^5^) and **35** (ZnL^7^) have a higher activity for the recognition of (acac)^−^ than Zn^2+^ complexes of tetradentate ligands.

#### 2.1.6. Stopped-Flow Experiments to Determine the Rates of ZnL-(acac)^−^ Complexation

Herein, stopped-flow experiments were performed to more-precisely determine the rates of formation of ZnL-(acac)^−^ complex. The increase in UV/Vis absorption of acac (0.2 mM) with **33** (l-ZnL^5^, 3 mM) at 294 nm was monitored and a rate constant of 6.18 (±0.03) × 10^−2^ s^−1^ was calculated from the resulting curve by fitting to a single exponential equation. Similar studies of the complexation between acac and **45** (l-ZnL^13^) and **49** (l-ZnL^17^) afforded rate constants of 9.03 (±0.07) × 10^−2^ s^−1^ and 7.47 (±0.05) × 10^−2^ s^−1^, respectively, which were almost the same as that of **33** (l-ZnL^5^). These formation rates of ZnL-(acac)^−^ complex are about 1.4 × 10^5^ higher than that for enaminone **55**.

#### 2.1.7. Comparison between Reactivity of Enamine and Zn^2+^-Enolate Intermediates

**Figure 4.**
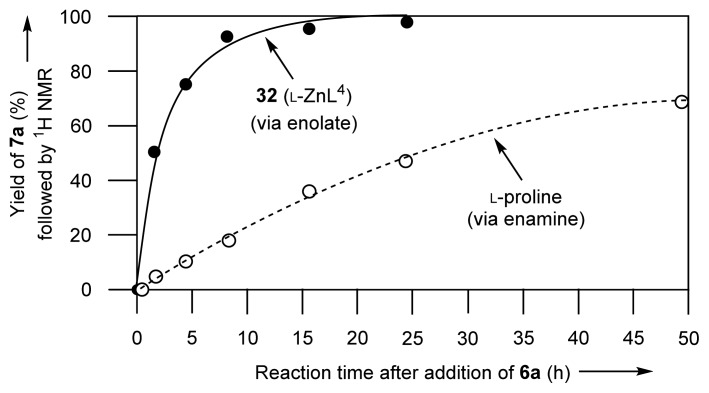
Change in the yield of **7a** followed by ^1^H-NMR in the presence of **32** (l-ZnL^4^) (plain curve) and l-proline (dashed curve).

**Scheme 11.**
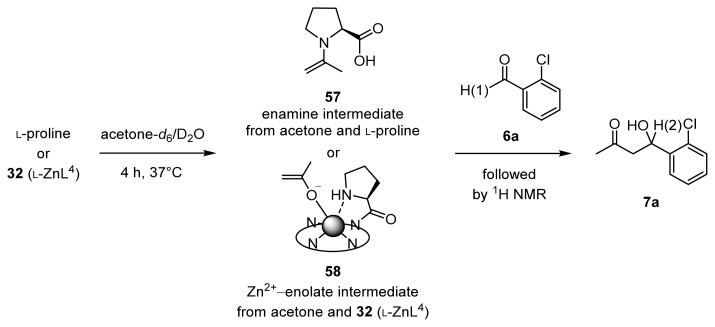
Comparison of the reactivity of Zn^2+^-enolate and enamine intermediates by ^1^H-NMR spectroscopy.

The reactivity of an enamine intermediate **57** (from acetone-*d*_6_ and l-proline) and a Zn^2+^-enolate intermediate **58** (from acetone-*d*_6_ and 50 mM **32** (l-ZnL^4^)), in D_2_O at 37 °C, were compared by ^1^H-NMR spectroscopy (Scheme 11). The aldol reaction of the enamine intermediate **57** with 2-chlorobenzaldehyde **6a** had not gone to completion even after 50 h (dashed curve in Figure 4). On the other hand, in the presence of **32** (l-ZnL^4^), it was found that **6a** was converted quantitatively in 24 h at 37 °C (plain curve in Figure 4). The initial reaction rate of the aldol reaction between acetone and **6a** via **58** is approximately 10 times higher than that via **57**, indicating that the Zn^2+^-enolate is more reactive than enamine species.

#### 2.1.8. X-ray Crystal Structure of **48** (l-ZnL^16^)

**Figure 5.**
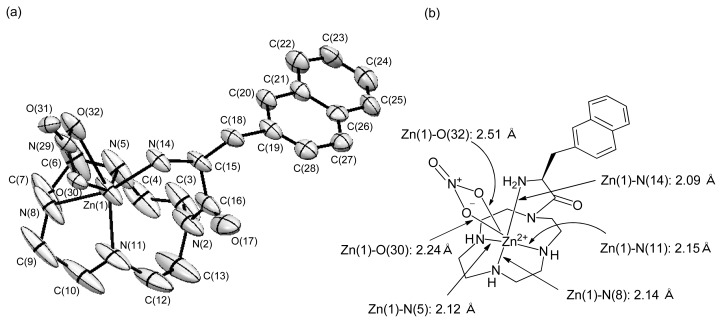
(**a**) ORTEP (Oak Ridge Thermal Ellipsoid Plot) of drawing of **48** (l-ZnL^16^(NO_3_)_2_); thermal ellipsoids are set at 50% probability; (**b**) Coordination-bond lengths in **48** (l-ZnL^16^(NO_3_)_2_). A Zn^2+^ ion is 6-coordinated by these nitrogen atoms of a cyclen ring, one nitrogen atom of the side chain, and two oxygen atoms of the NO_3_ anion. All of the hydrogen atoms and one NO_3_ anion are omitted for clarity.

Initially, it was assumed that the NH_2_ group of the amino acid moiety would coordinate weakly or not at all to Zn^2+^ center (Scheme 8). X-ray crystal structure analyses of **48** (l-ZnL^16^) disclosed that the Zn^2+^ was coordinated not only by three nitrogen atoms (N(5), N(8), and N(11)) of cyclen and a NO_3_ anion, but also by the nitrogen atom (N(14)) of β-naphthylalanyl moiety, as shown in Figure 5. The approximate Zn–N bond lengths are 2.12 Å for Zn–N(5), 2.14 Å for Zn–N(8), 2.15 Å for Zn–N(11), and 2.09 Å for Zn–N(14), thus, implying that these Zn–N coordinate-bond lengths are almost identical. In this structure, the NO_3_ anion coordinates to Zn^2+^ atom and the bond lengths for the two Zn–O (NO_3_^−^) coordinate-bonds are about 2.24 Å (Zn–O(30)) and about 2.51 Å (Zn–O(32)). It is very likely that this Zn^2+^-bound NO_3_ anion is replaced by H_2_O in aqueous solution, based on our previous findings [23,24]. The Zn^2+^ center in **50** (l-ZnL^18^) was also coordinated by the nitrogen atom of diphenylalanyl moiety and by one water molecule [35].

#### 2.1.9. Proposed Mechanism for the Aldol Reaction of Acetone Catalyzed by Chiral Zn^2+^ Complexes

**Scheme 12.**
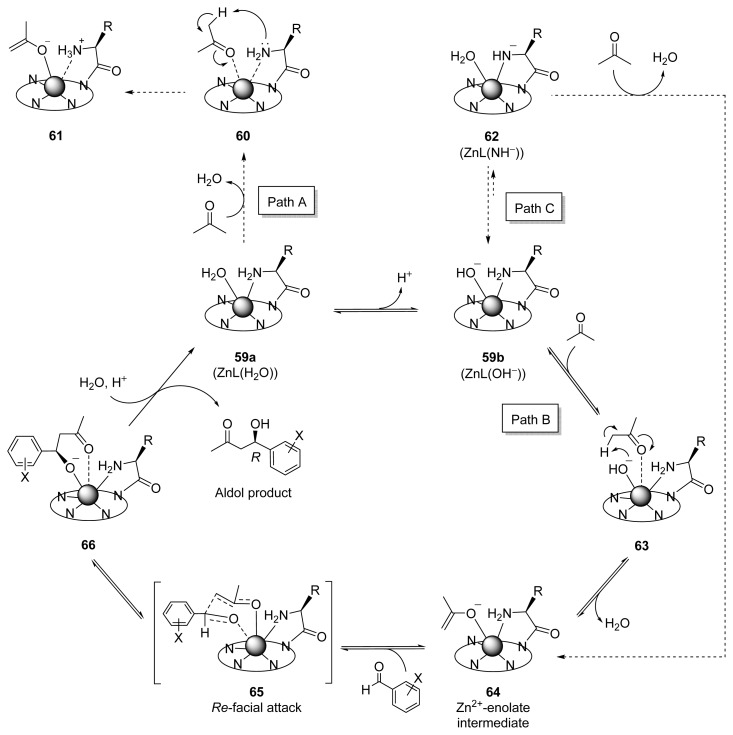
Proposed mechanism for the aldol reaction of acetone catalyzed by chiral Zn^2+^ complexes.

Proposed reaction mechanisms for the ZnL-catalyzed aldol reactions of acetone and benzaldehydes **6** based on the aforementioned results are shown in Scheme 12. Our initial hypothesis involved path A, in which the amine group of the side chain in Zn^2+^ complexes deprotonated the α-proton of acetone, which was activated by coordination to the Lewis acidic Zn^2+^ center, as shown in **60**, to generate the Zn^2+^-enolate complex **61**. However, given the data reported herein, this pathway appears to be less plausible, as the basicity of the amino side chain would be lowered by its coordination to the Zn^2+^ center, as observed in the X-ray crystal structure (Figure 5).

Herein, two other possibilities were considered, namely, paths B and C. It has been reported that Zn^2+^-bound HO^−^ and alkoxide species can act as bases and nucleophiles [23,24]. As shown in Scheme 7, the Zn^2+^-bound HO^−^ in the class II aldolase model complex **27b** (ZnL^2^(OH^−^)) is considered to deprotonate the α-proton to the carbonyl group of the phenacyl side chain to give the Zn^2+^-enolate form **27c** (Zn(H_−1_L^2^)) in aqueous solution [25]. Accordingly, it is hypothesized in path B that the OH^−^ of **59b** deprotonates the α-proton of acetone with the aid of the Lewis acidic Zn^2+^ in **63** and generates the Zn^2+^-enolate intermediate **64**.

Path C shows another possibility, in which the Zn^2+^-bound OH^−^ deprotonates the NH_2_ group to give rise to a NH^−^ species **62**, which deprotonates acetone, thus resulting in the formation of Zn^2+^-enolate intermediate **64**. However, this is unlikely because the p*K*_a_ value of the amine group would be over 30, which is much higher than those of Zn^2+^-bound H_2_O and alcohol moieties in typical Zn^2+^-cyclen complexes (p*K*_a_ value: 7~9) [23,24].

A consideration of these points allows us to conclude that path B is the most plausible among the three possibilities, although we do not completely rule out the path A scenario. Then, Zn^2+^-enolate intermediate **64** reacts with an acceptor aldehyde through six-membered transition state **65** to afford **66**, from which the aldol product is released and **59** is regenerated. The enantioselectivity could be explained in terms of the Zimmerman-Traxler-type transition state **65** [36]. We assume that the Zn^2+^ in **65** is 5- (or 6-) coordinated by four nitrogen atoms of ligand, the enolate of the acetone, and the carbonyl group of the aldehyde, by analogy with the 5- (or 6-)coordinated Zn^2+^ in **32**-(acac)^−^ complex **56** in Scheme 10, and that enolate predominantly attacks at the *Re*-face of the aldehydes.

### 2.2. Asymmetric Aldol Reactions of Hydroxylated Acetones and Cyclic Ketones

#### 2.2.1. Asymmetric Aldol Reactions of Hydroxylated Acetones (Hydroxyacetone **4** and Dihydroxyacetone **14**)

Aldol reactions of unprotected α-hydroxyketones such as hydroxyacetone (HA) **4** and dihydroxyacetone (DHA) **14**, were examined (Tables 6 and 7). This approach would provide effective methods for the synthesis of 1,2-diol derivatives and, hopefully carbohydrate derivatives. The reaction between **4** and 4-nitrobenzaldehyde **6b** (in THF/**4**/H_2_O, DMSO/**4**/H_2_O, and **4**/CH_3_CN) in the presence of **32** (l-ZnL^4^) gave **67** in good yield, but with poor enantioselectivities (entries 1–3). In these reactions, the *anti*-stereoisomer was obtained as the major product. On the other hand, in the presence of **33** (l-ZnL^5^) containing a primary amine unit, the enantioselectivity in favor of **67** was improved (entries 4–6). When a **4**/CH_3_CN solvent system was employed, **33** (l-ZnL^5^) gave the aldol product *syn*-selectively (*anti*/*syn* = 37/63) in 91% yield and with 45% *ee* (3*S*, 4*R*) (entry 7).

Table 7 shows the results for the aldol reaction between **14** and **6b** to give the triol aldol adduct, which was immediately acetylated to afford **16** in order to determine its stereoselectivity. As listed in entries 1 and 4, **32** (l-ZnL^4^) and **33** (l-ZnL^5^) poorly facilitated the aldol reaction in THF/H_2_O (H_2_O is required for the solvation of both substrates). In the DMSO/H_2_O solvent system, which gave a homogeneous reaction mixture, **32** (l-ZnL^4^) and **33** (l-ZnL^5^) afforded **16** in 22% and 27% yields, respectively, but with poor enantioselectivities (entries 2 and 5). When the NMP/H_2_O solvent system was used, *syn*-selectivity was observed (*anti*/*syn* = 26/74 and 34/66) (entries 3 and 6) with moderate enantioselectivities (44% *ee* (3*R*, 4*S*)) in favor of *syn*-**16** (entry 6).

Whereas **32** (l-ZnL^4^) afforded the aldol products *anti*-**67**, from **4** and **6b**, and *syn*-**16**, from **14** and **6b**, with poor enantioselectivities, **33** (l-ZnL^5^) gave the *syn* forms of **67** and **16** with moderate enantioselectivities (Tables 6 and 7). These results suggest that a primary amine unit on the side chain is required for enantiodiscrimination in aldol reactions between α-hydroxyketones, such as **4** and **14** and aldehydes [16]. It was assumed that the enantioselectivity in aldol reactions of **14** is influenced by two hydroxyl groups.

#### 2.2.2. Asymmetric Aldol Reactions of Cyclic Ketones (Cyclohexanone **9** and Cyclopentanone **68**)

Next, the aldol reaction of cyclohexanone **9** and **6b** was carried out using **45** (l-ZnL^13^) (5 mol %, 25 mM) in different solvent systems, as listed in entries 1–10 of Table 8. Although only trace amounts of aldol product **11** were obtained under neat conditions (entry 1), the reaction was accelerated in the case of **9**/H_2_O (95/5) to give **11** in 79% yield with 89% *ee* (2*R*, 1′*R*), while the *anti*/*syn* ratio was almost 1:1 (entry 2). When 5 mol % TFA was added, the diastereo- and enantioselectivity of *syn*-**11** were improved (entry 3) [37,38] (as was confirmed by ^1^H-NMR that **45** (l-ZnL^13^) did not decompose under these conditions). Among the alcohol solvents tested (MeOH, EtOH, and 2-propanol), MeOH gave *anti*-**11** in 72% *ee* (2*S*, 1′*R*) with an *anti*/*syn* ratio of 82/18 (entries 4–6). The use of aprotic polar solvents, such as NMP and DMF, gave *anti*-**11** as the major isomer (*anti*/*syn* = 90/10) with good enantioselectivities, although the chemical yields were rather low (entries 7 and 8). When a **9**/NMP/H_2_O mixture was used, the reaction proceeded smoothly to afford **11** in 85% yield with 83% *ee* (2*R*, 1′*R*) and an *anti*/*syn* ratio of ca. 1/1 (entry 9). On the other hand, *anti*-**11** was obtained as the major isomer with good diastereo- and enantioselectivity (*anti*/*syn* = 88/12, 84% *ee* (2*S*, 1′*R*)) when **9**/NMP/MeOH was used as the solvent (entry 10). A similar dependency of the stereochemistry of the aldol products on the solvent system used was observed when **49** (l-ZnL^17^) was used (entries 11 and 12). In entries 13 and 14, *syn*-**69** was obtained as a major isomer in a **68**/H_2_O system in high yield and with good enantioselectivities (entry 13), while the yield and stereoselectivities of **69** were low in **68**/NMP/MeOH (entry 14).

### 2.3. One-Pot Chemoenzymatic Synthesis of Chiral 1,3-Diols Using an Enantioselective Aldol Reaction and Enzymatic Reduction

#### 2.3.1. Introduction for One-Pot Chemoenzymatic Synthesis

Chemoenzymatic synthesis by the combined use of chemical catalysis and biocatalysis is a powerful methodology for the multistep synthesis of biologically important compounds, drugs, and so on. Organic reactions by artificial catalysts are generally conducted in organic solvents, as many organic molecules, such as reagents, catalysts, and intermediates, are usually not stable or soluble in water. In contrast, enzymatic reactions in living systems are conducted in aqueous solvents, because most of enzymes are functional only within a narrow range of temperature and pH levels and not so stable in less polar organic environments. This discrepancy makes it difficult to conduct one-pot chemoenzymatic reactions in an organic environment. Recently, the development of bioorthogonal reactions are growing as chemical reactions that neither interact with nor interfere with a biological system under physiological conditions [40] and it could be applied to chemoenzymatic synthesis.

**Scheme 13.**
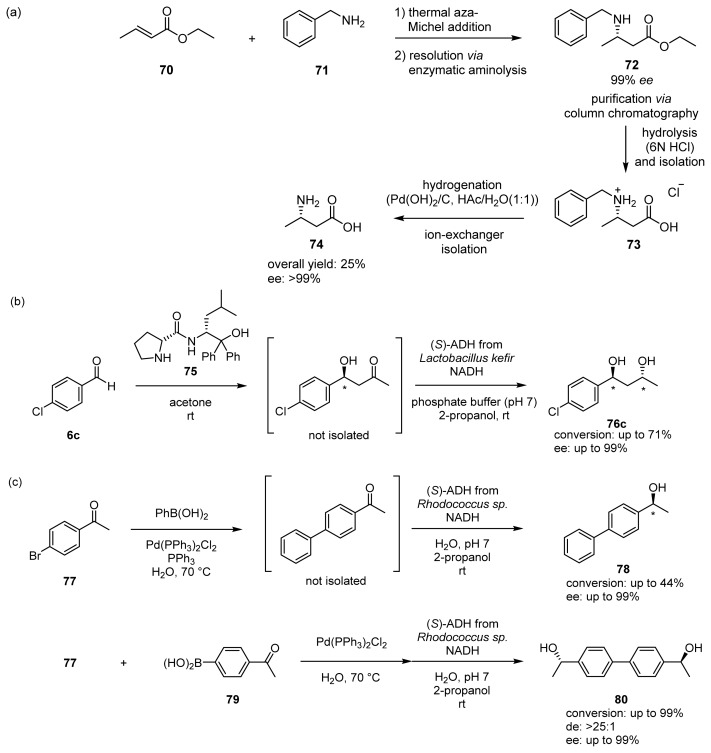
(**a**) Sequential and modular synthesis of enantiomerically pure β-amino acids; (**b**) Sequential and modular synthesis of chiral 1,3-diols with two stereogenic centers; (**c**) Combination of a palladium-catalyzed cross-coupling with an asymmetric biotransformation.

To date, a number of examples of one-pot processes have been developed, based on chemocatalytic tandem reactions, multienzymatic reactions, and biotechnological reactions [41–43]. It is assumed that one-pot processes involving chemical catalysts and biocatalysts take full advantage of the productivity of chemical catalysts and the chemo-, region-, and stereoselectivity of biocatalysts [2]. For example, Gröger *et al*. reported on an enantioselective synthesis of ethyl (*S*)-3-aminobutanoic acid **74** by means of combinations of aza-Michel reaction between **70** and **71**, and the kinetic resolution via aminolysis catalyzed by *Candida antarctica* lipase to give **72**, followed by hydrolysis and hydrogenation (Scheme 13a) [44]. Chiral 1,3-diols derivatives of aromatic compounds such as **76** were synthesized by enantioselective aldol reactions of acetone with **6c** using organocatalysts **75** and successive enantioselective reduction of ADH and NADH system (Scheme 13b) [45]. Chiral biaryl-containing alcohols, **78** and **80**, were synthesized in very high optically yields using a combination of cross-coupling reactions of **77** with **79** promoted by palladium catalysts and enantioselective enzymatic reduction (Scheme 13c) [46].

To date, numerous publications for the stereoselective synthesis of 1,3-diols that contain two stereogenic centers and important interemediate for various purposes have been reported [1,47], involving asymmetric homogeneous and heterogeneous hydrogenation and diastereoselective reduction [48], radical chain elongation [49], enzymatic and non-enzymatic asymmetrization [50,51], dynamic kinetic resolution [52], and stereoselective aldol-Tishchenko reactions [53]. However, there is still substantial demand for stereoselective synthetic methods to produce all possible stereoisomers of chiral 1,3-diols.

**Scheme 14.**
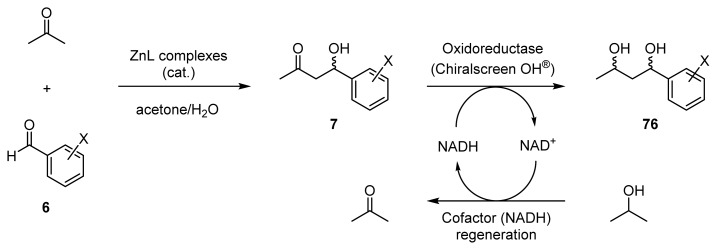
One-pot synthesis of optically active 1,3-diols **76** by chemoenzymatic synthesis in an aqueous solvent, involving enantioselective aldol reactions of acetone with **6** catalyzed by chiral Zn^2+^ complexes (ZnL) and the successive enzymatic reduction of **7** with regeneration of the NADH cofactor.

It was expected that the one-pot chemoenzymatic synthesis by the combined use of chiral Zn^2+^ catalysts and enzymes in an aqueous solvent would be useful for the selective synthesis of all of the possible stereoisomers of 1,3-diols **76** in a one-pot manipulation involving enantioselective aldol reactions of acetone with benzaldehydes to give **7** using chiral Zn^2+^ complexes, and the successive enantioselective reduction of **7** to give **76** using oxidoreductases with the regeneration of the NADH (reduced form of nicotinamine adenine dinucleotide) cofactor (Scheme 14) [54].

#### 2.3.2. Enantioselective Reductions of β-Hydroxyketones **7a**–**c** Using Oxidoreductase

For the reduction of β-hydroxyketones **7a**–**c**, we first chose Baker’s yeast alcohol dehydrogenase (ADH) and oxidoreductases from *Saccharomyces cerevisiae* (*S. cerevisiae*) and *Lactobacillus kefir* (*L. kefir*) as these enzymes have been reported to catalyze the enantioselective reduction of 4-phenyl-4-hydroxy-2-butanone [55]. However, Baker’s yeast ADH and *S. cerevisiae* ADH were not very effective for the reduction of **7a** (entries 1 and 2 in Table 9). In entry 3, it was found that the ADH from *L. kefir* is effective for the stereoselective reduction of **7a**, albeit this enzyme produces only the (*R*)-form of **76a**.

We, thus, decided to test the “Chiralscreen^®^ OH” kit, which is available from Daicel Co., Ltd, Niigata, Japan, and contains a library of recombinant NADH-dependent oxidoreductases [56]. Generally, oxidoreductases require an equivalent amount of NAD(P)H (reduced form) for activity. The reductases in “Chiralscreen^®^ OH” themselves can reduce NAD^+^ (oxidized form) to NADH using 2-propanol as a hydride source, so that the concentration of NADH can be reduced to a catalytic amount. It has been also reported that “Chiralscreen^®^ OH” can be used to catalyze the reduction of a variety of ketone even if the solubility of the substrate is low in aqueous solution [56].

The results of the stereoselective reduction of racemic **7a**–**c** using “Chiralscreen^®^ OH” enzymes in 100 mM phosphate buffer (pH 7.2) at 30 °C were listed in entries 4–12. Among the nine enzymes of “Chiralscreen^®^ OH” tested (E001, 021, 031, 039, 041, 051, 057, 092, and 119), four enzymes such as E001, E031, E039, and E092, were found to be effective for the reduction of **7a** (entries 4–7) [34]. In general, E001 and E039 gave better chemical yields than E031 and E092 (entries 4 and 6 *versus* entries 5 and 7). Interestingly, it was found that E001 and E039 give (*S*)- and (*R*)-forms of **76a** (99% *ee*), respectively, with respect to the stereogenic center (position 3) in **76a**. It should also be noted that the *anti*/*syn* ratios of **76a** were almost 1:1, thus indicating that kinetic resolution negligibly occurred (except for E092 in entry 7). It was also found that **7b** and **7c** are converted into **76b** and **76c** by E001 and E039, respectively (entries 8–11), and the reduction of (*S*)-**7a** (90% *ee*) with E001 exclusively gave *anti*-**76a** (1*S*, 3*S*) as the main product (entry 12).

#### 2.3.3. One-Pot Chemoenzymatic Synthesis of Optically Active 1,3-Diols **76a**–**c** from Acetone and Benzaldehydes **6a**–**c**

**Scheme 15.**
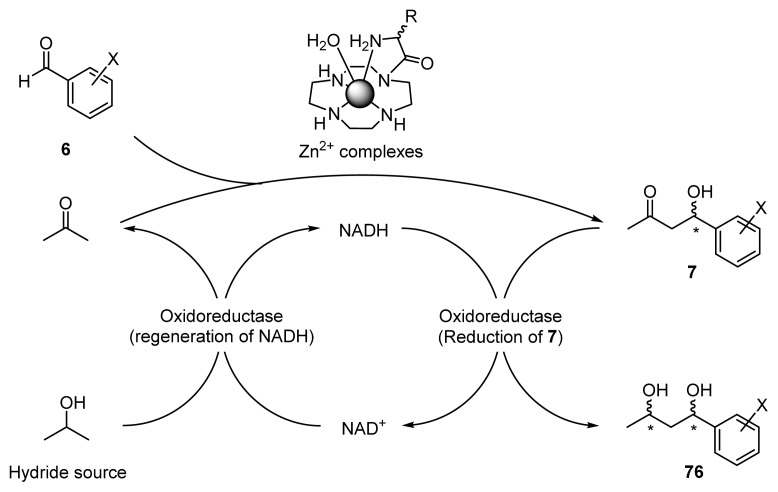
Scheme of initially attempted one-pot synthesis of optically active 1,3-diols **76** by chemoenzymatic synthesis in an aqueous solvent.

To examine the one-pot synthesis of 1,3-diols **76a**–**c** from acetone and benzaldehydes **6a**–**c**, simultaneous one-pot aldol-reduction reactions was attempted in a mixture containing acetone, **6a**–**c**, Zn^2+^ complexes (**36** (l-ZnL^8^) and **37** (d-ZnL^8^)), NADH, and “Chiralscreen^®^ OH”-E001 or E039 (Scheme 15). It was assumed that acetone, which would be generated as a byproduct of the regeneration of NADH from 2-propanol and NAD^+^, could be used as the substrate of aldol reaction, so that the amount of acetone could be reduced and the aldol-reduction cycle could proceed in just one reactor. However, this system did not work, because an excess amount of acetone was required to promote the aldol reaction, during which the Chiralscreen^®^ enzymes were inactivated. As a result, only aldol product **7** was obtained with the negligible formation of **76**.

**Scheme 16.**
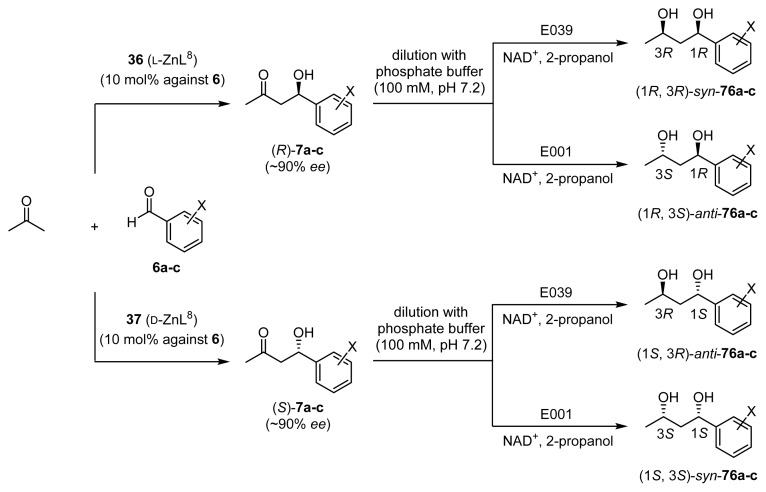
Summary of the one-pot chemoenzymatic synthesis of 1,3-diols **76a**–**c** from acetone and benzaldehydes **6a**–**c**.

Then, step-wise chemoenzymatic reaction was performed, as summarized in Scheme 16. The enantioselective aldol reaction between acetone and **6** with **36** (l-ZnL^8^) or **37** (d-ZnL^8^) (10 mol %) was conducted in acetone/H_2_O to give **7**. The reaction mixture was then diluted with phosphate buffer (100 mM, pH 7.2), and the enzyme, NAD^+^, and 2-propanol were added for the reduction of the aldol product **7**.

The results are also listed in Table 10. The aldol reaction of **6a** with acetone in the presence of **36** (l-ZnL^8^) and the successive reduction by E001 gave **76a** in 88% yield with a *syn*/*anti* ratio of approximately 4/96 ((1*R*, 3*S*)-**76a** is a major isomer; as listed in entry 1). Employing E039 instead of E001 switched the product to (1*R*, 3*R*)-**76a** (entry 2). The use of **37** (d-ZnL^8^) with E001 and E039 gave (1*S*, 3*S*)-**76a** and (1*S*, 3*R*)-**76a**, respectively, with >95% *ee* (entries 3 and 4). These results suggest that all four stereoisomers of 1,3-diols can be prepared by selecting the appropriate chiral Zn^2+^ complexes and oxidoreductases. Similarly, entries 5–8 and 9–12 indicate that 4-nitrobenzaldehyde **6b** and 4-chlorobenzaldehyde **6c** can be converted into all possible stereoisomers of the corresponding 1,3-diols, **76b** and **76c**.

## 3. Conclusions

We reviewed the design and synthesis of chiral Zn^2+^ complexes comprising chiral amino acids and Zn^2+^-cyclen complexes, inspired by two classes of natural aldolases. The combined findings indicate that these Zn^2+^ complexes are efficient catalysts for asymmetric aldol reactions of acetone with benzaldehydes in water-containing solvent systems and that Zn^2+^ complexes that contain appropriate hydrophobic and bulky side chains give high chemical and optical yields (up to 97% yield and 96% *ee*). Mechanistic studies including UV/Vis titrations of ZnL with acac and X-ray crystal structure analysis of the Zn^2+^ complexes indicate that these catalysts accelerate the aldol reactions via a Zn^2+^-enolate intermediate, which is generated by the cooperative functions of Zn^2+^ ion of Zn^2+^ complexes that activate ketone substrates as Lewis acids and the Zn^2+^-bound OH^−^ that deprotonates the α-proton of ketones.

One of the advantages of these aldol reactions is that they are applicable to the one-pot synthesis of biorelevantly important compounds such as the optically active 1,3-diol **76** by using a combination of the enantioselective aldol reactions catalyzed by chiral Zn^2+^ complexes and successive reduction by the recombinant oxidoreductase system “Chiralscreen^®^ OH”. Typical examples include the one-pot chemoenzymatic synthesis from acetone and **6a** with **36** (l-ZnL^8^) and E001 to afford (1*R*, 3*S*)-**76a** in 88% yield with 96% *ee*. Using these methodologies, all of the possible stereoisomers of **76a**–**c** can be obtained when the appropriate ZnL aldol catalyst and oxidoreductases are used.

We conclude that these results afford useful information concerning the design, synthesis, and mechanistic study of new artificial catalysts for catalytic enantioselective aldol reactions. Further catalyst design promises to lead, not only to the development of efficient stereoselective reactions, but also to the development of more practical and useful chemoenzymatic and biocompatible synthesis methods.

## Figures and Tables

**Table 1.** Results for an asymmetric aldol reaction between acetone and **6a** catalyzed by l-proline and Zn^2+^ complexes.

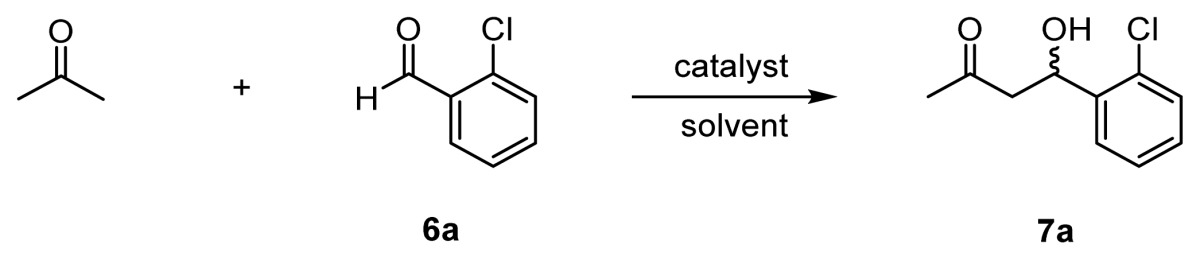							

Entry	Catalyst [a] (mM)	Mol %	Solvent [b]	Conditions	Yield [c] (%)	CTN [d]	*ee* (%) [e]
1	l-proline (20)	20	DMSO/acetone	4 h, 25 °C	85	4	67 (*R*)
2	**31** (l-ZnL^3^) [f] (10)	10	DMSO/acetone	26 h, 25–50 °C	3	–	1 (*R*)
3	**32** (l-ZnL^4^) [f] (10)	10	DMSO/acetone	4 h, 25 °C	12	1	34 (*R*)
4	l-proline (50)	5	acetone/H_2_O	20 h, 37 °C	22	4	48 (*R*)
5	**31** (l-ZnL^3^) [f] (50)	5	acetone/H_2_O	20 h, 37 °C	43	9	1 (*R*)
6	**32** (l-ZnL^4^) [g] (50)	5	acetone/H_2_O	20 h, 37 °C	73	15	80 (*R*)
7	**29** (l-L^4^) [h] (50)	5	acetone/H_2_O	20 h, 37 °C	72	14	*racemic*
8	**41a** (l-CdL^4^) [f] (50)	5	acetone/H_2_O	20 h, 37 °C	5	1	50 (*R*)
9	**41b** (l-CuL^4^) [f] (50)	5	acetone/H_2_O	20 h, 37 °C	trace	–	–
10	**33** (l-ZnL^5^) [f] (50)	5	acetone/H_2_O	20 h, 37 °C	87	17	80 (*R*)
11	**34** (l-ZnL^6^) [f] (50)	5	acetone/H_2_O	20 h, 37 °C	54	11	9 (*R*)
12	**35** (ZnL^7^) [g] (50)	5	acetone/H_2_O	20 h, 37 °C	5	1	2 (*S*)
13	**36** (l-ZnL^8^) [f] (50)	5	acetone/H_2_O	24 h, 25 °C	85	17	91 (*R*)
14	**37** (d-ZnL^8^) [f] (50)	5	acetone/H_2_O	24 h, 25 °C	85	17	91 (*S*)
15	**26** (ZnL1) [g] (50)	5	acetone/H_2_O	20 h, 37 °C	trace	–	–
16	l-proline + **26** (ZnL^1^) [g] (50)	5	acetone/H_2_O	20 h, 37 °C	trace	–	–

a Numbers in parentheses are the concentrations of catalysts in solvent;

b Solvent ratio: DMSO/acetone = 4/1 (entries 1–3), acetone/H_2_O = 4/1 (entries 4–16);

c Isolated yield;

d Catalytic Turnover Number (=yield/equivalents of catalyst);

e Determined by HPLC analysis using a chiral column (references [31,34]);

f Zn^2+^ complexes, l-CdL^4^, and l-CuL^4^ were formed *in situ*;

g Isolated Zn^2+^ complexes were used;

h l-L^4^ was extracted with CHCl_3_ from aq. NaOH (pH > 12) prior to use.

**Table 2.** Results for asymmetric aldol reactions between acetone and benzaldehydes (**6b**–**e**) catalyzed by **32** (l-ZnL^4^), **33** (l-ZnL^5^), **36** (l-ZnL^8^), and **37** (d-ZnL^8^).

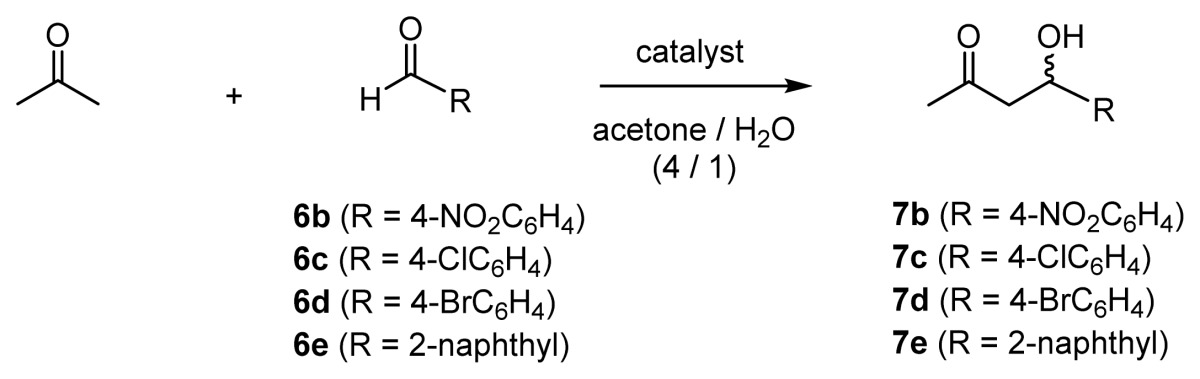								

Entry	Substrate	Catalyst [a]	mol %	Conditions	Product	Yield (%) [b]	CTN [c]	*ee* (%) [d]
1	**6b**	**32** (l-ZnL^4^)	5	20 h, 37 °C	**7b**	78	16	63 (*R*)
2	**6b**	**33** (l-ZnL^5^)	5	20 h, 37 °C	**7b**	86	17	86 (*R*)
3	**6b**	**36** (l-ZnL^8^)	10	24 h, 30 °C	**7b**	quant	10	90 (*R*)
4	**6b**	**37** (d-ZnL^8^)	10	24 h, 30 °C	**7b**	quant	10	90 (*S*)
5	**6c**	**36** (l-ZnL^8^)	10	72 h, 30 °C	**7c**	quant	10	90 (*R*)
6	**6c**	**37** (d-ZnL^8^)	10	72 h, 30 °C	**7c**	quant	10	90 (*S*)
7	**6d**	**32** (l-ZnL^4^)	10	20 h, 37 °C	**7d**	70	7	52 (*R*)
8	**6d**	**33** (l-ZnL^5^)	10	20 h, 37 °C	**7d**	49	5	75 (*R*)
9	**6e**	**32** (l-ZnL^4^)	10	120 h, 37 °C	**7e**	59	6	57 (*R*)
10	**6e**	**33** (l-ZnL^5^)	10	120 h, 37 °C	**7e**	55	6	83 (*R*)

a Concentrations of catalysts in the solvent were 50 mM. Zn^2+^ complexes were formed *in situ*;

b Isolated yield;

c Catalytic turnover number (=chemical yield/equivalents of catalyst);

d Determined by HPLC analysis using a chiral column (references [31,34]).

**Table 3.** Results for an asymmetric aldol reaction between acetone and **6a** catalyzed by **42**–**54** (l-ZnL^10^–ZnL^22^) in acetone/H_2_O.

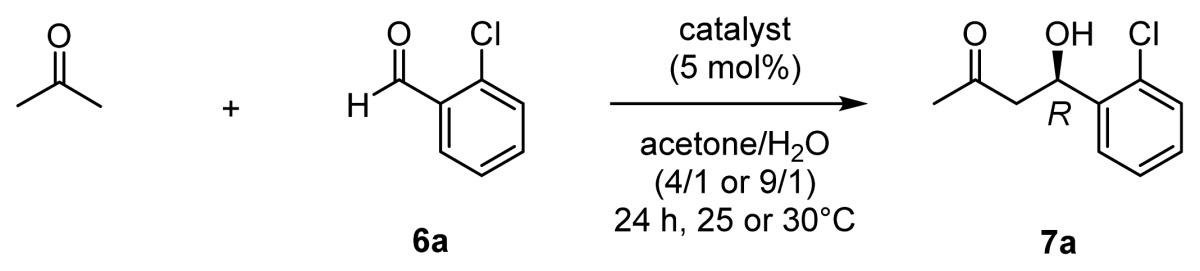				

Entry [a]	Catalyst [b]	Yield (%) [c]	CTN [d]	*ee* (%) [e]
1	**42** (l-ZnL^10^)	30	6	91 (*R*)
2	**43** (l-ZnL^11^)	89	18	94 (*R*)
3	**44** (l-ZnL^12^)	84	17	86 (*R*)
4	**45** (l-ZnL^13^)	91	18	94 (*R*)
5	**46** (l-ZnL^14^)	67	13	87 (*R*)
6	**47** (l-ZnL^15^)	86	17	78 (*R*)
7	**48** (l-ZnL^16^)	93	19	93 (*R*)
8	**49** (l-ZnL^17^)	95	19	94 (*R*)
9	**50** (l-ZnL^18^)	54	11	88 (*R*)
10	**51** (l-ZnL^19^)	44	9	78 (*R*)
11	**52** (l-ZnL^20^)	79	16	92 (*R*)
12	**43** (l-ZnL^11^)	90	18	96 (*R*)
13	**45** (l-ZnL^13^)	85	17	95 (*R*)
14	**49** (l-ZnL^17^)	96	19	96 (*R*)
15	**53** (ZnL^21^)	24	5	9 (*R*)
16	**54** (ZnL^22^)	19	4	33 (*S*)

a Reaction conditions: acetone/H_2_O = 4/1, 24 h, 30 °C (Entries 1–11), acetone/H_2_O = 9/1, 24 h, 25 °C (Entries 12–14), and acetone/H_2_O = 4/1, 24 h, 25 °C (Entries 15–16);

b Concentrations of catalysts in the solvent were 50 mM. Zn^2+^ complexes were formed *in situ*;

c Isolated yield;

d Catalytic Turnover Number (=yield/equivalents of catalyst);

e Determined by HPLC analysis using a chiral column (reference [35]).

**Table 4.** Results for asymmetric aldol reactions between acetone and benzaldehydes (**6b**–**c**, and **6f**–**g**) catalyzed by **45** (l-ZnL^13^) and **49** (l-ZnL^17^) in acetone/H_2_O.

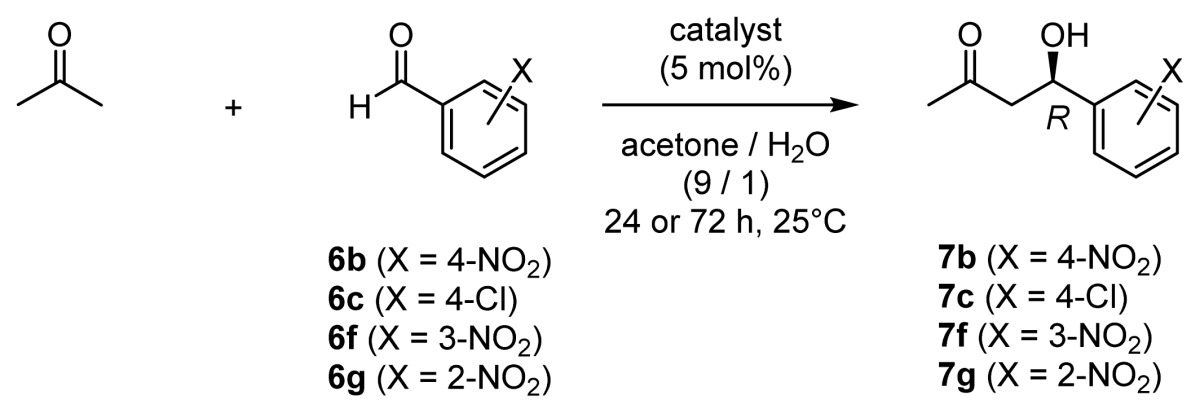						

Entry [a]	Substrate	Catalyst [b]	Product	Yield (%) [c]	CTN [d]	*ee* (%) [e]
1	**6b**	**45** (l-ZnL^13^)	**7b**	92	18	96 (*R*)
2	**6b**	**49** (l-ZnL^17^)	**7b**	92	18	95 (*R*)
3	**6c**	**45** (l-ZnL^13^)	**7c**	74	15	94 (*R*)
4	**6c**	**49** (l-ZnL^17^)	**7c**	83	17	95 (*R*)
5	**6f**	**45** (l-ZnL^13^)	**7f**	92	18	95 (*R*)
6	**6f**	**49** (l-ZnL^17^)	**7f**	97	19	95 (*R*)
7	**6g**	**45** (l-ZnL^13^)	**7g**	96	19	90 (*R*)
8	**6g**	**49** (l-ZnL^17^)	**7g**	89	18	92 (*R*)

a Reaction conditions: 24 h, 25 °C (Entries 1, 2, and 5–8), 72 h, 25 °C (Entries 3 and 4);

b Concentrations of catalysts in the solvent were 50 mM. Zn^2+^ complexes were formed *in situ*;

c Isolated yield;

d Catalytic Turnover Number (=yield/equivalents of catalyst);

e Determined by HPLC analysis using a chiral column (reference [35]).

**Table 5.** The apparent enaminone or Zn^2+^-enolate formation constants (*K*_app_) for l-proline, l-valine, Zn(OTf)_2_, **32** (l-ZnL^4^), **33** (l-ZnL^5^), **35** (ZnL^7^), and **26** (ZnL^1^) with acetylacetone (acac) in DMSO/H_2_O (1/2) at 25 °C (λ_max_ and *K*_app_ are absorption maxima and apparent formation constants of the catalyst-(acac) complexes, respectively).

	l-proline	l-valine	Zn(OTf)_2_	32 (l-ZnL^4^)	33 (l-ZnL^5^)	35 (ZnL^7^)	26 (ZnL^1^)
**λ_max_**	316 nm (enaminone)	316 nm (enaminone)	289 nm (Zn^2+^-enolate)	294 nm (Zn^2+^-enolate)	292 nm (Zn^2+^-enolate)	295 nm (Zn^2+^-enolate)	–
***K*_app_**	~10 (62 [a])	~10	157	212	139	589 (49 [b])	n. i. [c]

a The *K*_app_ value in DMSO/H_2_O (95/5);

b The *K*_app_ value obtained by potentiometric pH titration in H_2_O with *I* = 0.1 (NaNO_3_) at 25 °C (p*K*_a_ of acac = 8.9);

c Negligible interaction with acac.

**Table 6.** Results for an asymmetric aldol reaction between **4** and **6b** catalyzed by **32** (l-ZnL^4^) and **33** (l-ZnL^5^).

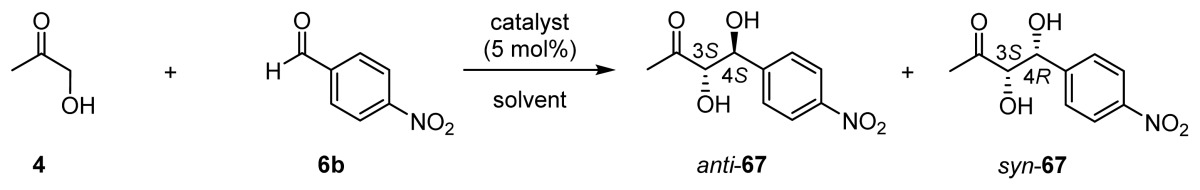							

Entry	Catalyst [a] (mM)	Solvent	Conditions	Yield [b] (%)	CTN [c]	d.r. [d] (*anti*/*syn*)	*ee* [%] [e] (*anti*/*syn*)
1	**32** (l-ZnL^4^) (25)	THF/**4**/H_2_O (5/4/1)	24 h, 37 °C	83	17	75/25	*racemic*/*racemic*
2	**32** (l-ZnL^4^) (50)	DMSO/**4**/H_2_O (21/1.1/1)	20 h, 37 °C	80	16	52/48	*racemic*/4 (3*S*, 4*R*)
3	**32** (l-ZnL^4^) (25)	**4**/CH_3_CN (1/1)	24 h, 25 °C	90	18	75/25	1 (3*R*, 4*R*)/8 (3*S*, 4*R*)
4	**33** (l-ZnL^5^) (25)	THF/**4**/H_2_O (2/1/1)	20 h, 37 °C	89	18	64/36	7 (3*S*, 4*S*)/5 (3*S*, 4*R*)
5	**33** (l-ZnL^5^) (25)	**4**/H_2_O (3/1)	20 h, 37 °C	68	14	58/42	9 (3*S*, 4*S*)/14 (3*S*, 4*R*)
6	**33** (l-ZnL^5^) (25)	**4**/NMP [f] (1/1)	24 h, 25 °C	96	19	63/37	18 (3*S*, 4*S*)/2 (3*R*, 4*S*)
7	**33** (l-ZnL^5^) (25)	**4**/CH_3_CN (1/1)	24 h, 25 °C	91	18	37/63	8 (3*R*, 4*R*)/45 (3*S*, 4*R*)

a Numbers in parentheses are the concentrations of catalysts in solvent. Zn^2+^ complexes were formed *in situ*;

b Isolated yield;

c Catalytic Turnover Number (=yield/equivalents of catalyst);

d Determined by ^1^H-NMR spectroscopy and HPLC analysis;

e Determined by HPLC analysis using a chiral column (reference [31]);

f NMP = *N*-methylpyrrolidone.

**Table 7.** Results for an asymmetric aldol reaction between **14** [a] and **6b** catalyzed by **32** (l-ZnL^4^) and **33** (l-ZnL^5^).

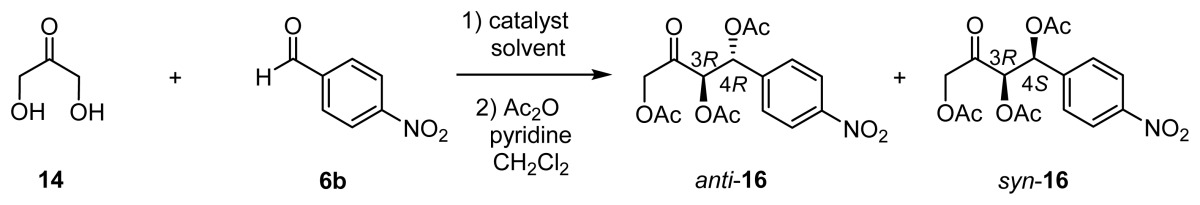							

Entry	Catalyst [b] (mM)	mol %	Solvent	Conditions (for aldol)	Yield [%] [c] (for 2 steps)	d.r. [d] (*anti*/*syn*)	*ee* [%] [e] (*anti*/*syn*)
1	**32** (l-ZnL^4^) (12)	5	THF/H_2_O (1/1)	96 h, 37 °C	Trace	–	–
2	**32** (l-ZnL^4^) (6)	5	DMSO/H_2_O (3/1)	72 h, 37 °C	22	37/63	*racemic*/6 (3*R*, 4*S*)
3	**32** (l-ZnL^4^) (100)	10	NMP/H_2_O (33/1)	24 h, 25 °C	4	26/74	3 (3*R*, 4*R*)/2 (3*S*, 4*R*)
4	**33** (l-ZnL^5^) (17)	5	THF/H_2_O (3/5)	72 h, 37 °C	4	42/58	3 (3*S*, 4*S*)/9 (3*R*, 4*S*)
5	**33** (l-ZnL^5^) (6)	5	DMSO/H_2_O (2/1)	72 h, 37 °C	27	37/63	*racemic*/6 (3*R*, 4*S*)
6	**33** (l-ZnL^5^) (100)	10	NMP/H_2_O (33/1)	42 h, 25 °C	26	34/66	8 (3*R*, 4*R*)/44 (3*R*, 4*S*)

a We used the DHA monomer (entries 1, 2, 4, and 5) or DHA dimer (entries 3 and 6). The equivalents of DHA (as monomer) relative to **6b** used were 3.4 (entry 1), 3 (entries 2 and 5), 5 (entry 4), and 2 equiv (entries 3 and 6);

b Numbers in parentheses are concentrations of catalysts in the solvent. Zn^2+^ complexes were formed *in situ*;

c Isolated yield;

d Determined by ^1^H-NMR spectroscopy and HPLC analysis;

e Determined by HPLC analysis using a chiral column (reference [31]).

**Table 8.** Results for asymmetric aldol reactions between cyclic ketones (**9** and **68**) and **6b** catalyzed by **45** (l-ZnL^13^) and **49** (l-ZnL^17^).

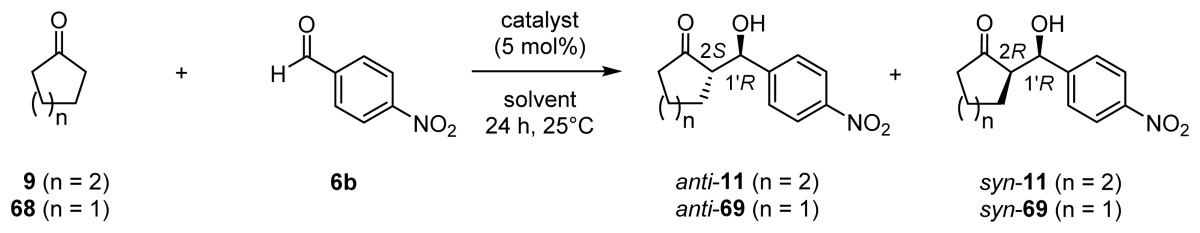							

Entry	Catalyst [a]	Solvent [b]	Product	Yield [c] (%)	CTN [d]	d.r. [e] (*anti*/*syn*)	*ee* [%] [f] (*anti*/*syn*)
1	**45** (l-ZnL^13^)	**9**	**11**	4	1	99/1	28 (2*S*, 1′*R*)/19 (2*R*, 1′*R*)
2	**45** (l-ZnL^13^)	**9**/H_2_O (95/5)	**11**	79	16	49/51	11 (2*S*, 1′*R*)/89 (2*R*, 1′*R*)
3	**45** (l-ZnL^13^) [g]	**9**/H_2_O (95/5)	**11**	67	13	30/70	2 (2*R*, 1′*S*)/92 (2*R*, 1′*R*)
4	**45** (l-ZnL^13^)	**9**/MeOH (50/50)	**11**	81	16	82/18	72 (2*S*, 1′*R*)/61 (2*R*, 1′*R*)
5	**45** (l-ZnL^13^)	**9**/EtOH (50/50)	**11**	61	12	67/33	68 (2*S*, 1′*R*)/86 (2*R*, 1′*R*)
6	**45** (l-ZnL^13^)	**9**/2-propanol (50/50)	**11**	13	3	80/20	68 (2*S*, 1′*R*)/54 (2*R*, 1′*R*)
7	**45** (l-ZnL^13^)	**9**/DMF (50/50)	**11**	4	1	91/9	84 (2*S*, 1′*R*)/52 (2*R*, 1′*R*)
8	**45** (l-ZnL^13^)	**9**/NMP (50/50)	**11**	19	4	90/10	88 (2*S*, 1′*R*)/17 (2*R*, 1′*R*)
9	**45** (l-ZnL^13^)	**9**/NMP/H_2_O (40/50/10)	**11**	85	17	49/51	7 (2*R*, 1′*S*)/83 (2*R*, 1′*R*)
10	**45** (l-ZnL^13^)	**9**/NMP/MeOH (40/50/10)	**11**	96	19	88/12	84 (2*S*, 1′*R*)/46 (2*R*, 1′*R*)
11	**49** (l-ZnL^17^)	**9**/H_2_O (95/5)	**11**	62	12	51/49	6 (2*S*, 1′*R*)/90 (2*R*, 1′*R*)
12	**49** (l-ZnL^17^)	**9**/NMP/MeOH (40/50/10)	**11**	89	18	87/13	83 (2*S*, 1′*R*)/51 (2*R*, 1′*R*)
13	**49** (l-ZnL^17^)	**68**/H_2_O (95/5)	**69**	90	18	31/69	23 (2*R*, 1′*S*)/87 (2*R*, 1′*R*)
14	**49** (l-ZnL^17^)	**68**/NMP/MeOH (40/50/10)	**69**	6	1	55/45	37 (2*R*, 1′*S*)/7 (2*R*, 1′*R*)

a Concentrations of catalysts in the solvent were 25 mM. Zn^2+^ complexes were formed *in situ*;

b Reaction solution is homogeneous in entries 1, 4–10, 12, and 14. Reaction solution is heterogeneous in entries 2–3, 11, and 13;

c Isolated yield;

d Catalytic Turnover Number (=yield/equivalents of catalyst);

e Determined by ^1^H-NMR spectroscopy and HPLC analysis;

f Determined by HPLC analysis using a chiral column (reference [39]);

g TFA (5 mol %) was used as a additive.

**Table 9.** Results for the asymmetric reduction of β-hydroxyketones **7a**–**c** catalyzed by oxidoreductase with NADH regeneration in 100 mM phosphate buffer (pH 7.2) at 30 °C for one day.

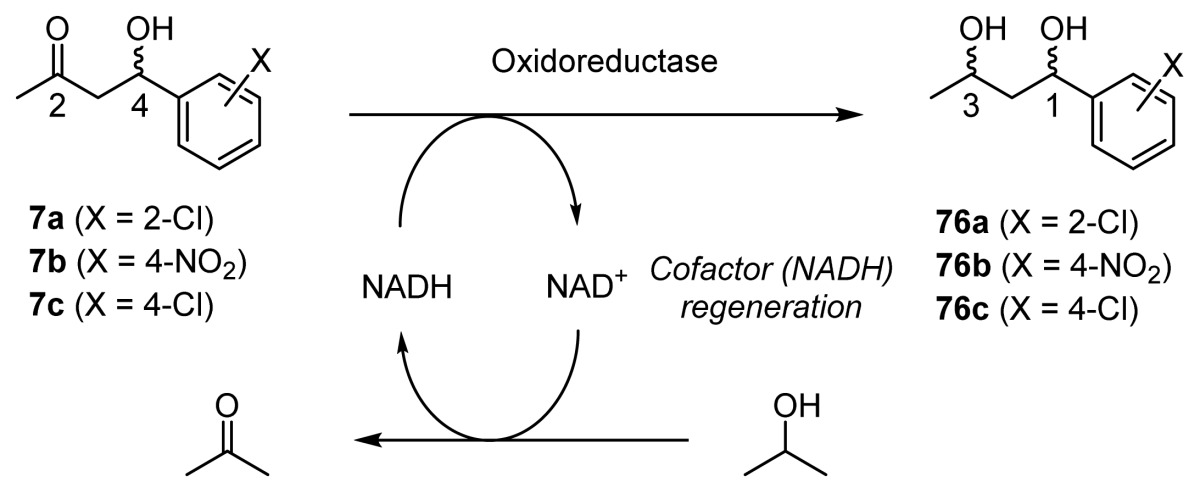						

Entry	Substrate	Oxidoreductase [a] (*syn*/*anti*)	Product [c]	Yield [%] [b] (*syn*/*anti*)	*ee* [%] [c] (*syn*/*anti*)	3*R*/3*S* [c]
1	*rac*-**7a** [d]	Baker’s yeast	–	trace	–	–
2	*rac*-**7a** [d]	ADH from *S. cerevisiae*	–	trace	–	–
3	*rac*-**7a** [d]	ADH from *L. Kefir*	**76a** (52/48)	quant	>99 (1*R*, 3*R*)/>99(1*S*, 3*R*)	>99/<1
4	*rac*-**7a** [d]	E001 [e]	**76a** (54/46)	quant	>99 (1*S*, 3*S*)/>99 (1*R*, 3*S*)	<1/>99
5	*rac*-**7a** [d]	E031 [e]	**76a** (47/53)	50	95 (1*S*, 3*S*)/93 (1*R*, 3*S*)	3/97
6	*rac*-**7a** [d]	E039 [e]	**76a** (48/52)	quant	>99 (1*R*, 3*R*)/>99 (1*S*, 3*R*)	>99/<1
7	*rac*-**7a** [d]	E092 [e]	**76a** (78/22)	64	>99 (1*S*, 3*S*)/94 (1*R*, 3*S*)	1/99
8	*rac*-**7b** [d]	E001 [e]	**76b** (50/50)	quant	>99 (1*S*, 3*S*)/>99 (1*R*, 3*S*)	<1/>99
9	*rac*-**7b** [d]	E039 [e]	**76b** (48/52)	quant	>99 (1*R*, 3*R*)/>99 (1*S*, 3*R*)	>99/<1
10	*rac*-**7c** [d]	E001 [e]	**76c** (50/50)	quant	>99 (1*S*, 3*S*)/>99 (1*R*, 3*S*)	<1/>99
11	*rac*-**7c** [d]	E039 [e]	**76c** (49/51)	quant	>99 (1*R*, 3*R*)/>99 (1*S*, 3*R*)	>99/<1
12	**7a** (90% *ee* (*S*))	E001 [e]	**76a** (95/5)	quant	>99 (1*S*, 3*S*)/>99 (1*R*, 3*S*)	<1/>99

a Conditions for the enzymatic reductions: [ketone] = 10 mM in 100 mM phosphate buffer, 24 h, 30 °C in the presence of 2-propanol (100 mM) and NAD^+^ (2 mM);

b Isolated yield;

c Determined by ^1^H-NMR and HPLC analysis using a chiral column (reference [34]);

d Racemate of **7** was used as the substrate;

e Enzyme of Chiralscreen purchased from Daicel Co., Ltd.

**Table 10.** Results for the one-pot chemoenzymatic synthesis of chiral 1,3-diols **76a**–**c** in aqueous solvent by the combined use of enantioselective aldol reactions catalyzed by chiral Zn^2+^ complexes and enantioselective reduction using “Chiralscreen^®^ OH”.

Entry	Substrate	ZnL [a]	Oxidoreductase [b] (“Chiralscreen^®^ OH”)	Product	Yield (%) [c]	Product ratio [d]			

						(1*R*,3*R*)	(1*S*,3*R*)	(1*R*,3*S*)	(1*S*,3*S*)
1	**6a**	**36** (l-ZnL^8^)	E001	**76a**	88	<1	<1	96	4
2	**6a**	**36** (l-ZnL^8^)	E039	**76a**	88	95	5	<1	<1
3	**6a**	**37** (d-ZnL^8^)	E001	**76a**	84	<1	<1	4	96
4	**6a**	**37** (d-ZnL^8^)	E039	**76a**	92	5	95	<1	<1
5	**6b**	**36** (l-ZnL^8^)	E001	**76b**	83	<1	<1	93	7
6	**6b**	**36** (l-ZnL^8^)	E039	**76b**	87	94	6	<1	<1
7	**6b**	**37** (d-ZnL^8^)	E001	**76b**	91	<1	<1	4	96
8	**6b**	**37** (d-ZnL^8^)	E039	**76b**	80	4	96	<1	<1
9	**6c**	**36** (l-ZnL^8^)	E001	**76c**	68	<1	<1	96	4
10	**6c**	**36** (l-ZnL^8^)	E039	**76c**	60	96	4	<1	<1
11	**6c**	**37** (d-ZnL^8^)	E001	**76c**	60	<1	<1	5	95
12	**6c**	**37** (d-ZnL^8^)	E039	**76c**	48	4	96	<1	<1

a Concentrations of catalysts for the aldol reaction were 50 mM. Zn^2+^ complexes were formed *in situ*;

b Conditions for reductions by enzymes: [ketone] = 10 mM in 100 mM phosphate buffer (pH 7.2), 24 h, 30 °C in the presence of 2-propanol (100 mM) and NAD^+^ (2 mM);

c Isolated yield;

d Determined by HPLC analysis of a mixture of all stereoisomers by column chromatography using a chiral column (reference [34]).
